# Unraveling cooperative interactions between complexed ions in dual-host strategy for cesium salt separation

**DOI:** 10.3762/bjoc.21.68

**Published:** 2025-04-29

**Authors:** Zhihua Liu, Ya-Zhi Chen, Ji Wang, Qingling Nie, Wei Zhao, Biao Wu

**Affiliations:** 1 Key Laboratory of Medicinal Molecule Science and Pharmaceutics Engineering, Ministry of Industry and Information Technology, School of Chemistry and Chemical Engineering, Beijing Institute of Technology, Beijing 102488, China

**Keywords:** anion binding, cesium extraction, dual-host strategy, ion-pair interaction, solid–liquid extraction

## Abstract

The dual-host strategy offers a straightforward approach to ion separation, yet the nature of cooperative interactions between receptor-complexed cations and anions remains poorly understood. In this study, we utilize 18-crown-6 as a cation receptor and a tripodal hexaurea receptor **L** as an anion receptor to extract cesium salts (chloride, nitrate, carbonate, sulfate, and phosphate) from the solid phase into chloroform. Remarkably, Cs_3_PO_4_ exhibits the highest extraction efficiency, driven by strong cooperative interactions involving ion-dipole coordination between Cs^+^ and carbonyl (C=O) groups, as well as direct ion-pairing interactions between 18-crown-6-complexed Cs^+^ and hexaurea-bound PO_4_^3−^. Single-crystal structural analysis corroborates these interactions, shedding light on the underlying mechanisms and providing valuable guidance for the rational design of advanced dual-host systems for selective ion separation.

## Introduction

Ion-pair interaction, defined as the electrostatic attraction between a positively charged cation and a negatively charged anion, is prevalent across various disciplines including biology, chemistry, materials science, and ion batteries [1–3]. Fundamental understanding of ion-pairing can help to regulate their roles and relevant applications in chemical catalysis, battery performance, and ion binding, transport and separation [4–8]. Building on the extensive research into anion and cation receptors within the realm of supramolecular chemistry [9–12], numerous heteroditopic ion-pair receptors have been elaborately developed [13–15]. These receptors, consisting of binding sites for both anions and cations within a single molecule, have facilitated advancements in ion-pair recognition. This progress has led to the development of ion separation utilizing ion-pair receptors [16–20], which eliminate the need for auxiliary reagents to balance overall charges compared to the use of individual anion or cation receptors [21]. An alternative approach for ion separation involves the combination of an anion receptor and a cation receptor, known as the dual-host strategy [22–24]. Unlike ion-pair receptors, this strategy avoids the intricate, multi-step synthesis required for designing and making new receptors, thereby saving considerable time. However, the selectivity of ion separation achieved through the dual-host strategy may not match that of ion-pair receptors, possibly due to less defined interactions between the receptor-complexed anions and cations.

**Figure 1:**
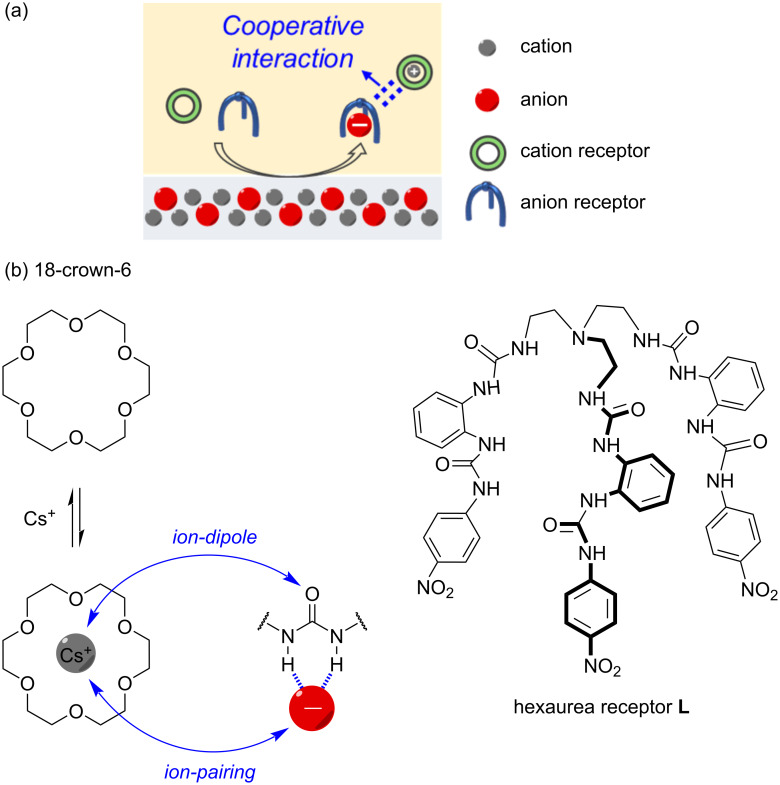
(a) Schematic illustration of the dual-host strategy for ion pair extraction via solid–liquid method, showing the cooperative interaction between complexed anion and complexed cation. (b) Molecular structures of 18-crown-6 (for Cs^+^ binding) and tripodal hexaurea receptor **L** (for anion binding), where cooperative interactions of ion-dipole and ion-pairing are shown.

Early studies employing the dual-host strategy were aimed at separating alkali metal halide salts from aqueous solutions into organic phases, including KCl, CsCl, and CsNO_3_ [23–29]. In these studies, 18-crown-6 was commonly utilized, and various anion receptors were selected to achieve tailored anion binding. Compared to the use of individual anion or cation receptors, the dual-host strategy can significantly enhance the efficiency of ion-pair extraction. However, the driving forces and cooperative interactions of the complexed ions remain poorly understood (Figure 1a). To the best of our knowledge, only two examples provide clear evidence of cooperative interactions based on single crystal structures [28–29], where the 18-crown-6 complexed K^+^ cation forms ion-dipole interactions with the carbonyl (C=O) or nitro (NO_2_) groups of the anion-bound receptors (KF and K_2_CO_3_).

Recently, we demonstrated that a tripodal hexaurea receptor **L** (Figure 1b) could selectively and reversibly extract sulfate and phosphate anions from water into organic phase (under pH control) [30–33]. Single crystal structures of the receptor–K_2_SO_4_ complex in the presence of 18-crown-6 clearly displayed ion-dipole interactions between K^+^ and C=O moieties [31], similar to these seen in the single crystal structures of KF and K_2_CO_3_ complexes. These provide a promising opportunity that can be used to identify the cooperative interaction underpinning complexed ions in dual-host strategy-based extraction. To do this, the hexaurea receptor, 18-crown-6 and Cs^+^ as cation were selected as model system, with the counter anion being varied from chloride, nitrate, carbonate, sulfate to phosphate. Solid–liquid extraction experiments and single-crystal structures demonstrated that the cooperative interactions (ion-dipole and ion-pairing) could be enhanced along with the charge of the anion and its binding affinity with **L** (from Cl^–^ to CO_3_^2–^, and PO_4_^3–^). Notably, for the first time, direct ion-pairing between receptor-complexed phosphate and 18-crown-6 complexed cesium was observed in the single crystal structure, facilitating highly efficient Cs_3_PO_4_ extraction.

## Results and Discussion

The tripodal hexaurea receptor **L** is comprised of a central tren (tris(2-aminoethyl)amine) core and three arms of *ortho*-phenylene bis(urea) units, which can fold inward to encapsulate an anion inside the cavity through up to 12 hydrogen bonds [30]. According to our previous results, the binding affinity of **L** with chloride, sulfate and phosphate is determined to be 2.2 × 10^2^ M^−1^, 9.9 × 10^4^ M^−1^, and 3.8 × 10^6^ M^−1^, respectively (in DMSO) [31]. Such strong anion binding affinity has led to selective extraction of sulfate and phosphate from basic aqueous solution into chloroform and controllable release into acidic solution [32]. Very recently, it was found that the receptor **L** alone can further extract solid Li_2_SO_4_ into DMSO solution [33], where sulfate binding is sufficiently strong to drive the solid–liquid extraction. DFT calculations suggest that an ion-dipole interaction of the Li^+^ cation and carbonyl groups also contribute to the extraction. The negative electrostatic potential (δ^−^) of O=C is attributed to a high dipole moment of the urea unit (mono(urea): 3.95 D, bis(urea): 7.55 D) [34–36], which has been demonstrated to be capable of binding Na^+^ and K^+^ by oligourea foldamers and macrocycles [37–39]. However, in the solid−liquid extraction of Li_2_SO_4_ in DMSO, addition of crown ether did not help to increase the extraction efficiency. This is because ion-dipole interactions are negligible in high polar solvent, and Li^+^ binding is weak [40–42]. Therefore, to further understand how the ion-dipole interactions regulate ion-pair separation, Cs^+^ was selected due to its relatively strong binding with 18-crown-6, >10^4^ M^−1^ in CH_3_CN, ≈10^3^ M^−1^ in DMSO [40,43–44]. Solid–liquid extraction is studied in chloroform as the ion-pairing interaction in nonpolar solvent could be stronger than that in polar solvent [45–48].

**Figure 2:**
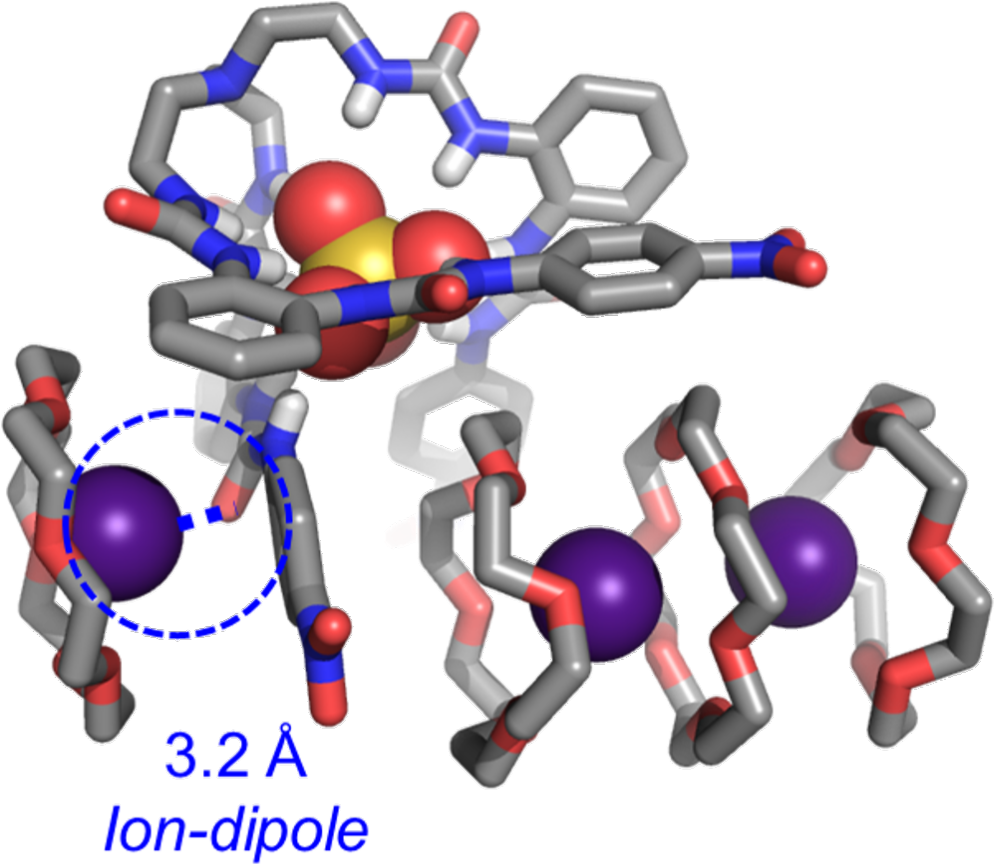
Single crystal structure of complexed Cs_2_SO_4_ with 18-crown-6 and tripodal receptor **L** (CCDC: 2411573). One sulfate anion is encapsulated inside the hexaurea cavity through 12 × N–H···O hydrogen bonds. One Cs^+^ cation is co-stabilized by electrostatic interactions with one 18-crown-6 and ion-dipole interactions with the O=C unit. Two Cs^+^ cations are complexed by two 18-crown-6 showing no interaction with anion receptor. The overall stoichiometry of Cs^+^, SO_4_^2−^, 18-crown-6 and the anion receptor is 4:2:5:2. Nonpolar hydrogen atoms and solvent molecules are omitted for clarity.

The ion-dipole interaction between complexed Cs^+^ cation and receptor–sulfate complex was first identified by single crystal structure analysis (Figure 2). The overall stoichiometry of **L**, 18-crown-6, Cs^+^, and SO_4_^2−^ was 4:5:4:2 in the crystalized structure. Like the structure of K_2_SO_4_ complexes [31], one Cs^+^ cation is encapsulated by 18-crown-6 and further stabilized by one ion-dipole interaction with the O=C unit of the hexaurea receptor. The Cs···O distance is measured at 3.2 Å. The other two Cs^+^ cations are found to be co-stabilized by three 18-crown-6 macrocycles.

Next, a solid–liquid extraction experiment was conducted for Cs_2_SO_4_ salts. A solution of hexaurea receptor **L** and two equivalents of 18-crown-6 in CHCl_3_ were prepared, and solid Cs_2_SO_4_ was added into the solution. Under stirring at 60 °C for 5 hours, all the solids were dissolved indicating the completion of solid–liquid extraction of Cs_2_SO_4._ In contrast, by changing the solvent from chloroform to acetonitrile, the Cs_2_SO_4_ solids were barely dissolved, consistent with weak Cs^+^ binding affinity with 18-crown-6 and negligible cooperative interaction between complexed ions in polar solvent of acetonitrile.

The solid–liquid extraction of other cesium salts including CsCl, CsNO_3_, Cs_2_CO_3_, and Cs_3_PO_4_ were further studied in the presence of one equivalent of hexaurea receptor versus anion and one equivalent of 18-crown-6 versus Cs^+^ cation. The best extraction efficiency was observed for Cs_3_PO_4_, where all the solids could be dissolved in CHCl_3_ in 0.5 h at 30 °C. Therefore, the extraction experiments were done at the same conditions (30 °C, 0.5 h, stirring rate: 1500 r/min), and the extraction results are summarized in Table 1. Specifically, the determined extraction efficiency (extracted Cs^+^ versus the initial amount; concentrations were recorded by ion chromatography) of CsCl, CsNO_3_, Cs_2_CO_3_, Cs_2_SO_4_ and Cs_3_PO_4_ were 82%, 36%, 61%, 41%, and 100%, respectively. In contrast, by using an individual 18-crown-6 or hexaurea receptor, a clear enhancement of extraction efficiency was seen for the dual-host strategy. Additionally, the use of 18-crown-6 alone displays better extraction efficiency than that of hexaurea receptor (Table 1), which is attributed to the relatively poor solubility of the hexaurea receptor in CHCl_3_.

**Table 1:** Summary of extraction efficiency and anion binding affinity.^a^

	Cation host only	Anion host only	Dual host	Anion binding affinity^b^ [M^−1^]	Anion hydration^c^ [kJ mol^−1^]

CsCl	68%	10%	82%	2.2 × 10^2^	−344
CsNO_3_	31%	13%	36%	3.3 × 10^3^	−286
Cs_2_CO_3_	39%	10%	61%	8.4 × 10^3^	−1324
Cs_2_SO_4_	16%	4%	41%	9.9 × 10^4^	−975
Cs_3_PO_4_	59%	5%	100%	3.8 × 10^6^	−2753

^a^Solid–liquid extraction conditions: 30 °C, 0.5 h, stirring rate: 1500 r/min, [L] = [anion] = ≈4.5 mM, chloroform. The extraction efficiency is defined as the extracted Cs^+^ over initial (total) Cs^+^ as determined by ion chromatography analysis. ^b^Anion binding affinities are determined by ^1^H NMR titration in DMSO-*d*_6_. Chloride, sulfate and phosphate binding affinities were reported in previous studies [31–32]. ^c^Gibbs energies of anion hydration at 25 °C. For Cs^+^, the hydration energy is −266 kJ mol^−1^ [49–50].

For cesium salts with various oxyanions, the extraction efficiency follows the order of PO_4_^3−^ > CO_3_^2−^ > SO_4_^2−^ > NO_3_^−^, consistent with the order of negative charges as well as the anions’ hydration energies (Table 1) [49–50]. This *anti*-Hofmeister selectivity of phosphate over other studied oxyanions is normally seen in liquid–liquid and solid–liquid anion extractions, likely due to relatively strong phosphate binding with hexaurea receptor (3.8 × 10^6^ M^−1^). In comparison, the binding affinity of the hexaurea receptor with nitrate and carbonate was calculated to be 3.3 × 10^3^ M^−1^ and 8.4 × 10^3^ M^−1^, respectively (vide infra), as determined by ^1^H NMR titrations in DMSO.

**Figure 3:**
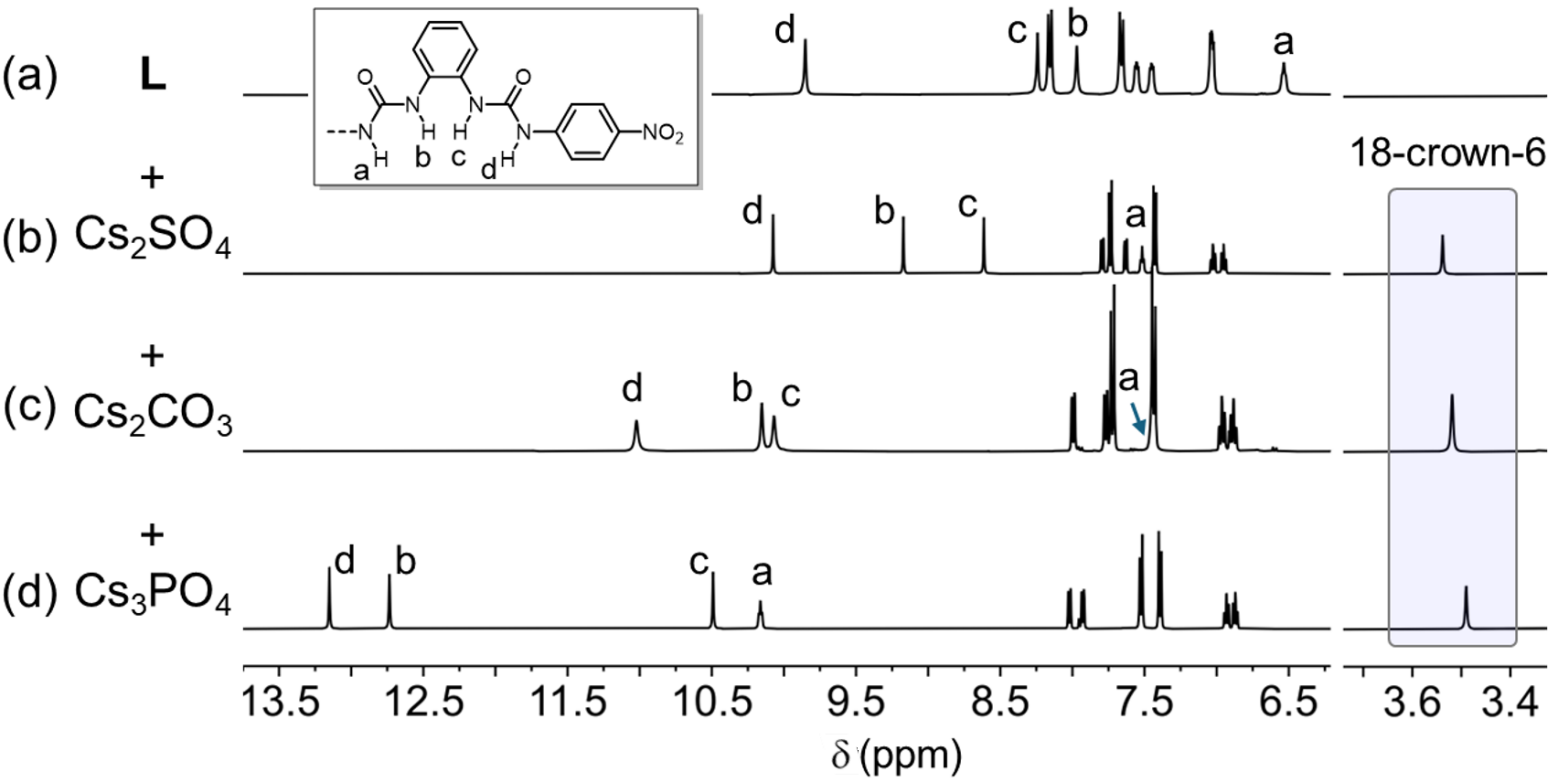
Stacked ^1^H NMR spectra of (a) free anion receptor **L** and its complexes with one equivalent of (b) Cs_2_SO_4_, (c) Cs_2_CO_3_, and (d) Cs_3_PO_4_ in the presence of 18-crown-6 (DMSO-*d*_6_, 1 mM, 400 MHz, 298 K).

The resulting complexes after solid–liquid extraction were also characterized by ^1^H NMR spectroscopy (Figure 3), showing consistent anion binding profiles. By comparing with free hexaurea receptor **L**, the chemical shifts of the urea units N–H in the obtained complexes are observed to be downfield-shifted indicative of anion binding. The relative peak positions of N–H (8.5–13.5 ppm, Figure 3) are consistent with their anion binding affinity and solid–liquid extraction efficiency, PO_4_^3−^ > CO_3_^2−^ > SO_4_^2−^. In addition, the chemical shifts of 18-crown-6 (3.4–3.6 ppm) are observed to be slightly upfield-shifted in comparison to that of the free crown ether, indicating Cs^+^ binding, consistent with their extraction efficiency. The relatively upfield shifted chemical shift for the Cs_3_PO_4_ complex may indicate strong cooperative interaction upon solid–liquid extraction.

To understand cooperative interactions between complexed ions, we tried to grow the crystal structure of studied salts. Fortunately, single crystals of Cs_2_CO_3_ and Cs_3_PO_4_ complexes were obtained by slow vapor diffusion from acetonitrile and diethyl ether. Notably, clearly stronger ion-dipole interactions of complexed carbonate and complexed phosphate are illustrated than those as seen in the single crystal structure of the Cs_2_SO_4_ complex. To our surprise, direct ion-pairing between receptor-complexed phosphate and 18-crown-6-complexed cesium is observed for the first time in single the crystal structure (vide infra).

**Figure 4:**
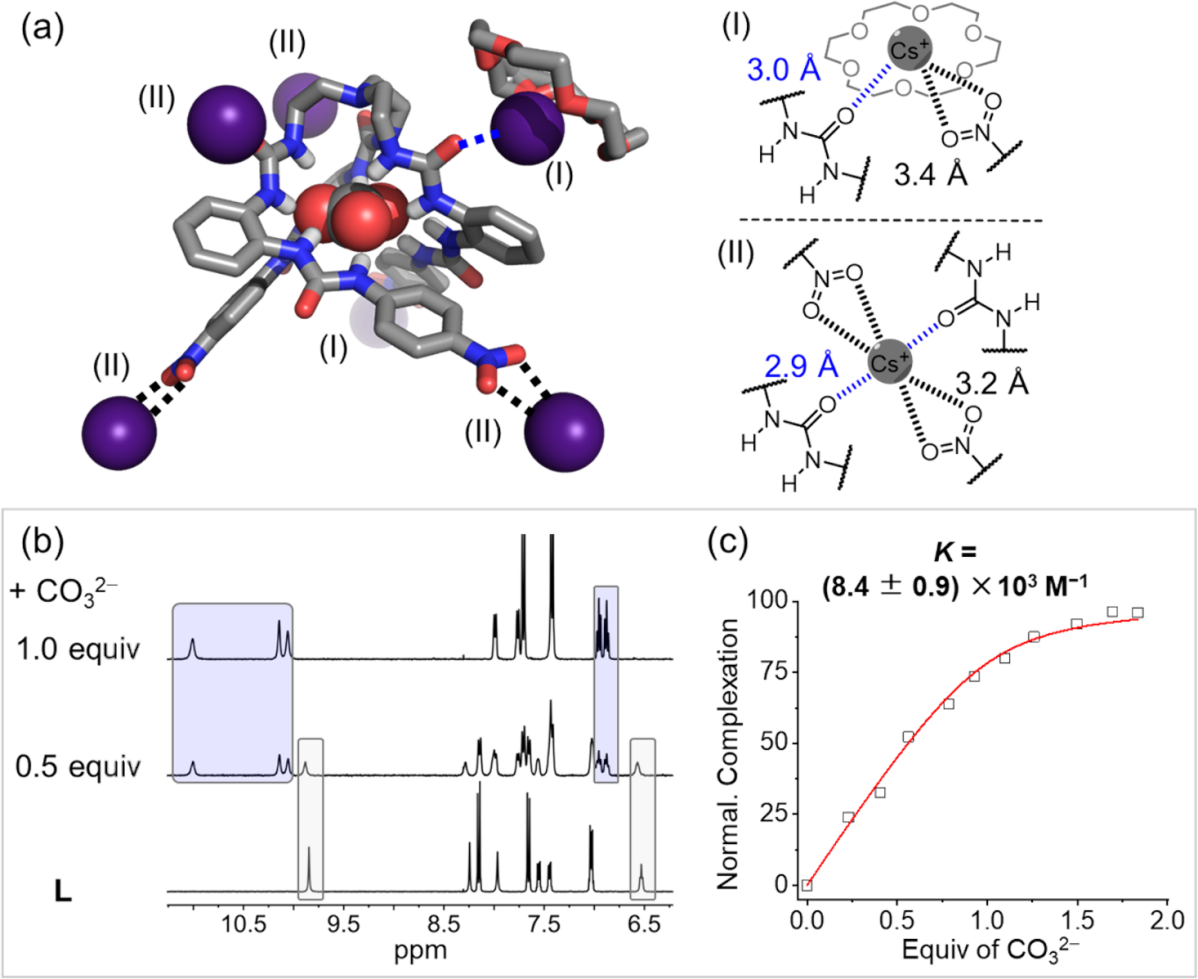
Single crystal structure of complexed Cs_2_CO_3_ (CCDC: 2411574) with 18-crown-6 and tripodal receptor **L**. The carbonate anion is encapsulated inside the hexaurea cavity and stabilized by 12 × N–H···O hydrogen bonds. Two types of Cs^+^ complexations are observed, (I) one 18-crown-6-complexed Cs^+^ interacts with carbonyl (O=C) and nitro (-NO_2_) groups through three ion-dipole interactions. (II) The other Cs^+^ is stabilized by six ion-dipole interactions from carbonyl (O=C) and nitro (-NO_2_) groups. The overall stoichiometry of Cs^+^, CO_3_^2−^, 18-crown-6 and anion receptor is 2:1:1:1. Nonpolar hydrogen atoms and solvent molecules are omitted for clarity. (b) Stacked ^1^H NMR spectra of free anion receptor **L** by adding carbonate (in the form of 18-crown-6 complexed Cs_2_CO_3_) showing slow-exchange of NMR signals (DMSO-*d*_6_, [**1**] = [salts] = 1 mM, 400 MHz, 298 K). (c) Fitted binding curve of carbonate complexation with receptor **L** as derived from NMR titration.

For the crystal of Cs_2_CO_3_ with 18-crown-6 and receptor **L** (Figure 4), an overall stoichiometry of one hexaurea receptor, one 18-crown-6, one carbonate and two cesium cations are obtained. Firstly, carbonate is encapsulated inside the folded cavity of the hexaurea receptor through twelve hydrogen bonds. The average distance of N···O is measured at 2.85 ± 0.08 Å, which is comparable to those that are seen in the single crystal structure of Cs_2_SO_4_ (average distance is 2.9 ± 0.07 Å). Based on an NMR titration of hexaurea receptor **L** by adding CO_3_^2−^, slow exchange of NMR signals is observed (Figure 4b), which is similar to that of sulfate anion titration results and indicative of a strong carbonate binding affinity. The carbonate binding constant is determined to be 8.4 ± 0.9 ×10^3^ M^−1^ in DMSO-*d*_6_ (Figure 4c), which is weaker than sulfate binding (9.9 × 10^4^ M^−1^). This is because that carbonate displays higher hydration energy than that of sulfate, and the tetrahedral shape of sulfate anion matches the pseudo-tetrahedral cavity of the folded hexaurea receptor [30].

Secondly, for Cs^+^ cations, two types of Cs^+^ binding are shown in the solid state. Two type-(I) cesium cations are found to be stabilized by the binding with 18-crown-6 and three ion-dipole interactions with O=C (urea unit) and O–N (terminal nitro group). Distances of Cs–O are measured at 3.0 Å and 3.4 Å. Regarding type-(II) cesium binding, four cesium cations are observed to interact with two urea units and two nitro groups through six Cs–O ion-dipole interactions (2.9 Å and 3.2 Å). These intermolecular interactions of Cs^+^ cations with18-crown-6 and hexaurea receptors help to form 3D framework in the solid state (Supporting Information File 1, Figure S2), which may reinforce cooperative interactions between complexed Cs^+^ and complexed CO_3_^2−^ for solid–liquid extraction. In contrast, only one ion-dipole interaction (3.2 Å) is observed in the single crystal structure of the Cs_2_SO_4_ complex. The enhanced ion-dipole interactions of complexed Cs_2_CO_3_ corresponds to relatively higher extraction efficiency (61%) than that of Cs_2_SO_4_ (41%).

**Figure 5:**
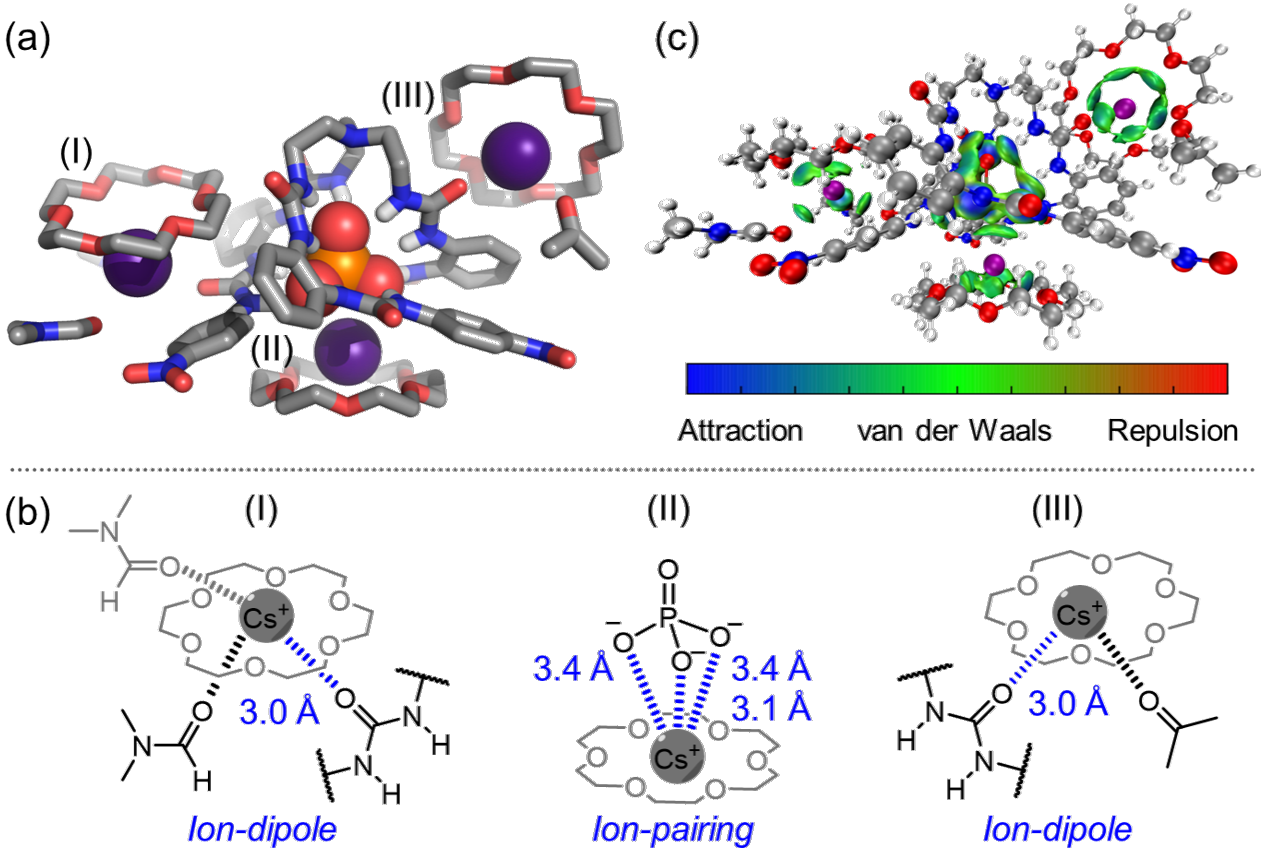
(a) Single crystal structure of complexed Cs_3_PO_4_ with 18-crown-6 and tripodal receptor (CCDC: 2411575) and (b) chemical structures for three types of intermolecular interactions with complexed Cs^+^. (c) IGM plot illustrating the attraction between complexed Cs^+^ and complexed phosphate. Color coding in the range of −0.5 < ρ sign(λ2) < 0.5 a.u. Atom colors: green or grey = C, white = H, blue = N, red = O, orange = S, and purple = Cs. For the crystal structure, the overall stoichiometry of Cs^+^, PO_4_^3−^, 18-crown-6 and anion receptor is 3:1:3:1. Phosphate anion is encapsulated inside the hexaurea cavity and stabilized by 12 × N–H···O hydrogen bonds. Three types of Cs^+^ complexations are observed. All Cs^+^ are seen to be complexed with 18-crown-6 and further stabilized by (I) ion-dipole interactions with DMF and carbonyl (O=C) group, (II) ion-pairing with receptor-complexed phosphate anion, and (III) ion-dipole interactions with acetone and carbonyl (O=C) group.

The cooperative interaction of complexed Cs^+^ with complexed phosphate is illustrated in single crystal structure (Figure 5). Like other anions, phosphate is also complexed inside the cavity of hexaurea receptor through twelve hydrogen bonds. The average N···O distance is 2.79 ± 0.03 Å, corresponding to strong phosphate binding (3.8 × 10^6^ M^−1^ in DMSO) [32]. All three Cs^+^ cations are encapsulated by 18-crown-6 yet further stabilized by different secondary interactions (Figure 5b). Specifically, the type-(I) and type-(III) Cs^+^ cations form three and two ion-dipole interactions with O=C (urea unit, DMF, or acetone molecules), respectively. For the type-(II) Cs^+^ cation, direct ion-pairing interaction with complexed phosphate anion is clearly illustrated, where average P···O distance is 3.3 ± 0.2 Å. These cooperative interactions were further analyzed and visualized by independent gradient model (IGM) analysis. Based on the IGM plots, we can clearly see the attraction between 18-crown-6-complexed Cs^+^ and the phosphate–hexaurea complex (Figure 5c and Supporting Information File 1, Figure S25). In contrast, such cooperative attraction is relatively weak for the Cs_2_SO_4_ complex (Supporting Information File 1, Figure S26). To the best of our knowledge, this is the first time that receptor-complexed anion and complexed Cs^+^ form ion-pairing as verified by a single crystal structure. These ion-pairings combined with multiple ion-dipole interactions support highly efficient solid–liquid extraction of Cs_3_PO_4_ over other cesium salts.

The cooperative interactions between receptor-complexed caesium and anions were also supported by enhanced Cs^+^ binding affinity in the presence of anion-binding receptors. ^1^H NMR titrations were conducted by adding cesium (as tetraphenylborate salts) into the solution of 18-crown-6 with/without the presence of one equivalent of anion-binding receptors in CDCl_3_. By fitting the chemical shift changes of 18-crown-6, the Cs^+^ binding constant of free 18-crown-6 is determined to be 17.7 ± 0.1 M^−1^ (Supporting Information File 1, Figure S9). In contrast, the Cs^+^ binding constants in the presence of phosphate, carbonate and sulfate complexes are determined to be 226.1 ± 12.4, 71.8 ± 2.2, and 48.2 ± 2.7 M^−1^, respectively (Supporting Information File 1, Figures S10–S15). These are consistent with the extraction efficiency and indicate positive cooperativity. The cooperative factors (*K*_anion_ versus *K*_free_) are determined to be 12.8, 4.1, and 2.72 for phosphate, carbonate and sulfate anion, respectively.

## Conclusion

In summary, by using a model system of 18-crown-6 and tripodal hexaurea anion receptor **L** for the solid–liquid extraction of cesium salts (with various conteranion, chloride, nitrate, carbonate, sulfate and phosphate) into chloroform, we demonstrate efficient extraction of Cs_3_PO_4_ solids. The extraction efficiency follows the order of Cs_3_PO_4_ > CsCl > Cs_2_CO_3_ > Cs_2_SO_4_ > CsNO_3_, reflecting the hydration energies and binding affinities of the corresponding anions. Notably, single-crystal structural analyses reveal that the extraction performance correlates with cooperative interactions between 18-crown-6-complexed Cs^+^ and hexaurea-bound anions. For the Cs_3_PO_4_ complex, direct ion-pairing interactions are identified for the first time. These findings highlight the accessibility of the dual-host strategy and suggest that cooperative interactions between receptor-complexed ions can be fine-tuned for selective ion separation. Ongoing work aims to explore diverse combinations of anion and cation receptors for targeted ion separation applications.

## Supporting Information

Deposition numbers 2411573 (Cs_2_SO_4_ complex), 2411574 (Cs_2_CO_3_ complex), and 2411575 (Cs_3_PO_4_ complex) contain the supplementary crystallographic data for this paper. These data are provided free of charge by the joint Cambridge Crystallographic Data Centre and Fachinformationszentrum Karlsruhe Access Structures service via https://www.ccdc.cam.ac.uk/.

File 1 Nuclear magnetic resonance (NMR), mass and X-ray diffraction data.

## Data Availability

All data that supports the findings of this study is available in the published article and/or the supporting information of this article.
