# Supplementary material for: Unraveling cooperative interactions between complexed ions in dual-host strategy for cesium salt separation
Source: Beilstein J Org Chem. 2025 Apr 29;21:845–53. doi: 10.3762/bjoc.21.68 (PMC12051461; doi:10.3762/bjoc.21.68)
Supplement: File 1 — Nuclear magnetic resonance (NMR), mass and X-ray diffraction data. [file Beilstein_J_Org_Chem-21-845-s001.pdf]

BEILSTEIN JOURNAL OF ORGANIC CHEMISTRY

Supporting Information

for

Unraveling cooperative interactions between complexed ions in dual-host strategy for cesium salt separation

Zhihua Liu, Ya-Zhi Chen, Ji Wang, Qingling Nie, Wei Zhao and Biao Wu

Beilstein J. Org. Chem. **2025,** *21,* 845–853. doi:10.3762/bjoc.21.68

Nuclear magnetic resonance (NMR), mass and X-ray diffraction data

License and Terms: This is a supporting information file under the terms of the Creative Commons Attribution License (https://creativecommons.org/licenses/by/4.0). Please note that the reuse, redistribution and reproduction in particular requires that the author(s) and source are credited and that individual graphics may be subject to special legal provisions.
The license is subject to the *Beilstein Journal of Organic Chemistry* terms and conditions: (https://www.beilstein-journals.org/bjoc/terms)

Table of contents:

S1. General information

S2. X-ray diffraction single crystal structures

S3. ^{1}H NMR titration studies

S4. Solid–liquid extraction experiments

S5. Mass spectrometry

S6. IGM analysis

S1. General information

All starting materials and solvents were ordered from commercial sources (Beijing InnoChem, Aladdin, Macklin Science & Technology Co., Ltd.) and used without further purification. ^{1}H and ^{13}C NMR spectra were recorded on a Bruker AVANCE AV II-400/500 MHz spectrometer at 298 K. ^{1}H NMR chemical shifts were reported relative to residual solvent peaks (^{1}H NMR: 2.50 ppm for DMSO-d_6, 7.26 ppm for $CDCl_3$;^{13}C NMR: 39.52 ppm for DMSO-d_6, 77.1 ppm for $CDCl_3$). High resolution mass spectrometry data were obtained by a Bruker micro TOF-Q II ESI-Q-TOF LC/MS/MS. Single crystal X-ray data were measured on a Bruker D8 Venture Photon II diffractometer. The anion concentration in aqueous solution was recorded by Shine ion chromatography (CIC-D100, China). All aqueous solutions were prepared by using ultrapure water (18.25 MΩ·cm).

The cesium phosphate salts are not commercially available and were prepared by acid-base reaction from corresponding cesium hydroxide and phosphoric acid. The phosphoric acid (H_3PO_4) was placed in a small vial and certain amounts of hydroxide was added. The mixture was diluted to 1 mL and the prepared solution was used directly. Cs_3PO_4 (water): H_3PO_4 was mixed with three equivalents of CsOH (40% wt, H_2O).

S2. X-ray diffraction single crystal structures

[Cs(18-crown-6)]$_2SO_4$ (12 μL or 18 μL, 0.625 mol/L, prepared by mixing Cs_2SO_4 with 2 equivalents of 18-crown-6) was added to a suspension of **L** (16 mg) in acetonitrile (1 mL) and acetone (1 mL). After stirring overnight at room temperature, the solution was centrifugated. The obtained clear solution was used for crystal growing. Slow vapor diffusion of diethyl ether into above-mentioned solution provided yellow crystals of L•18-crown-6•Cs_2SO_4 within one week.

[Cs(18-crown-6)]$_2CO_3$ (9 μL, 0.625 mol/L, prepared by mixing Cs_2CO_3 with 2 equivalents of 18-crown-6) was added to a suspension of **L** (12 mg) in acetonitrile (1 mL) and acetone (1 mL). After stirring overnight at room temperature, the solution was centrifugated. The obtained clear solution was used for crystal growing. Slow vapor diffusion of diethyl ether into the above-mentioned solution provided yellow crystals of L•18-crown-6•Cs_2CO_3 within one week.

[Cs(18-crown-6)]$_3PO_4$ (22 μL , 0.616 mol/L, prepared by mixing Cs_3PO_4 with 3 equivalents of 18-crown-6) was added to a suspension of **L** (12 mg) in acetonitrile (1 mL) and acetone (1 mL). After stirring overnight at room temperature, the solution was centrifugated. The obtained clear solution was used for crystal growing. Slow vapor

diffusion of diethyl ether into above-mentioned solution provided white crystals of L•18-crown-6•Cs_3PO_4 within one week.

Table S1. Crystal data details for obtained structures.

Complex	**L**•18-C-6•Cs_2SO_4	**L**•18-C-6•Cs_2CO_3	**L**•18-C-6•Cs_3PO_4
Empirical formula	$C_{78}H_{84}Cs_2N_{16}O_{31}S$	$C_{61}H_{72}Cs_2N_{16}O_{21}$	$C_{182}H_{268}Cs_6N_{36}O_{71}P_2$
CCDC	2411573	2411574	2411575
Formula weight	2039.49	3882.54	3882.54
Crystal system	Monoclinic	Monoclinic	Triclinic
Space group	$P2_1/n$	$P2_1/n$	P-1
a (Å)	15.3688(19)	19.0373(8)	15.4237(2)
b(Å)	21.192(3)	22.1519(9)	16.2180(2)
c(Å)	29.312(4)	19.0940(8)	22.8955(3)
α(deg)	90	90	77.3610(10)
β(deg)	104.965(5)	116.5470	88.8450(10)
γ(deg)	90	90	86.5290(10)
V (Å^3)	9223(2)	7203.2(5)	5577.95(13)
Z	4	4	1
T (K)	180	180	180
F(000)	4152.0	3312.0	2540.0
$D_{calc,}$g/cm^3	1.469	1.094	1.475
Total no. of data	17017	13284	19654
Completeness to θ	0.999	1.000	1.000
θ range	2.229-25.436	2.191-25.421	2.221-25
μ /mm^{-1}	0.900	1.094	1.081
Data/restraints/ Parameters	17017/88/1167	13284/51/909	19654/995/1406
GoF on F^2	1.027	1.041	1.081
R1	0.0510	0.0627	0.0384
wR2	0.061	0.1765	0.1042

Table S2. Hydrogen bonding information in the crystal structure of L • 18-C-6•Cs_2SO_4.

D-H···*A*	d (*D*-H)	d (H···*A*)	d (D···*A*)	∠*D*H*A*
N2-H2···O28	0.88	2.12	2.924	151
N3-H3···O30	0.88	2.05	2.872	155
N4-H4···O30	0.88	2.08	2.873	149
N5-H5···O31	0.88	2.43	2.766	103
N6-H6···O28	0.88	1.93	2.77	159
N7-H7···O39	0.88	2.16	2.941	148
N8-H8···O29	0.88	1.96	2.941	158
N9-H9···O30	0.88	2.18	2.941	151
N10-H10···O28	0.88	2.38	2.941	107
N11-H11···O28	0.88	1.9	2.941	164
N12-H12···O31	0.88	2.13	2.941	151

Table S3. Hydrogen bonding information in the crystal structure of L • 18-C-6•Cs_2CO_3.

D-H···*A*	d (*D*-H)	d (H···*A*)	d (D···*A*)	∠*D*H*A*
N2-H2···O19	0.88	2.12	2.924	151
N3-H3···O19	0.88	2.05	2.872	155
N4-H4···O21	0.88	2.08	2.873	149
N5-H5···O21	0.88	2.43	2.766	103
N7-H7···O21	0.88	1.93	2.77	159
N8-H8···O21	0.88	2.16	2.941	148
N9-H9···O20	0.88	1.96	2.798	158
N10-H10···O20	0.88	2.18	2.983	151
N12-H12···O20	0.88	2.38	2.765	107
N13-H13···O20	0.88	1.9	2.757	164
N14-H14···O19	0.88	2.13	2.936	151
N15-H15···O19	0.88	1.91	2.762	161

Table S4. Hydrogen bonding information in the crystal structure of L • 18-C-6•Cs_3PO_4.

D-H···*A*	d (*D*-H)	d (H···*A*)	d (D···*A*)	∠*DHA*
N1-H1···O6	0.88	1.98	2.776	151
N3-H3···O10	0.88	1.97	2.819	162
N4-H4···O4	0.88	1.89	2.759	170
N5-H5···O4	0.88	1.92	2.773	162
N6-H6···O8	0.88	1.92	2.795	170
N7-H7···O10	0.88	2.04	2.829	149
N8-H8···O6	0.88	1.85	2.716	168
N9-H9···O8	0.88	1.91	2.769	164
N10-H10···O4	0.88	2.02	2.833	153
N12-H12···O8	0.88	2.44	2.784	104
N14-H14···O6	0.88	2.03	2.847	153
N14-H14···O10	0.88	2.48	2.812	103
N16-H16···O10	0.88	2.05	2.84	148

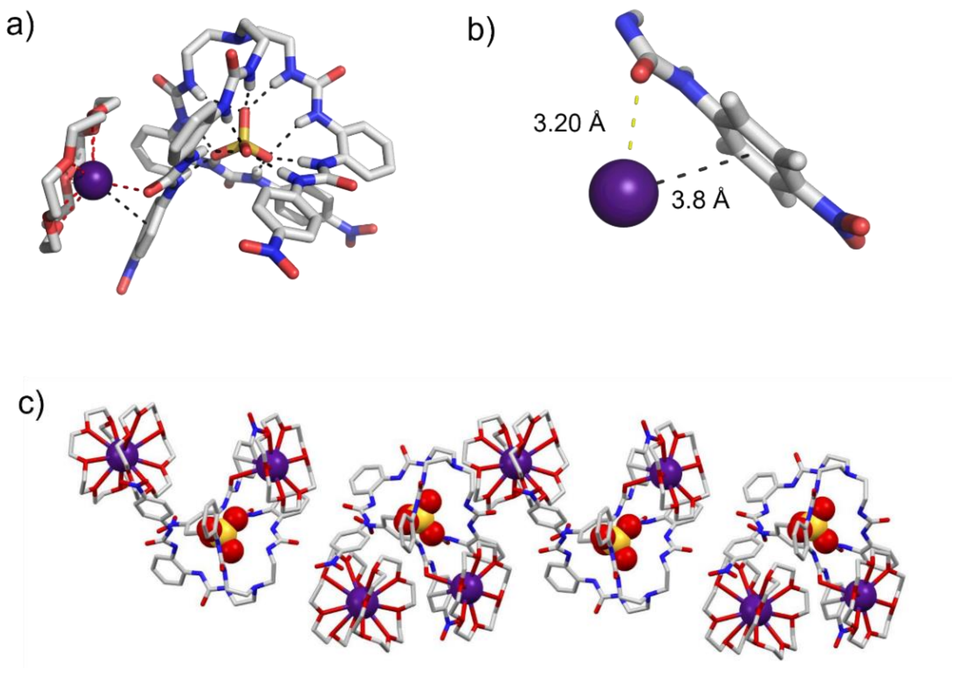

Figure S1. a) Single-crystal structure of the complex **L**•18-crown-6•Cs_2SO_4, the overall stoichiometry of Cs^+, SO_4^{2-}, 18-crown-6 and anion receptor is 4:2:5:2. b) Cs enhances structural stability by facilitating ion-π interactions and oxygen interactions with the urea-carbonyl moiety of the ligand. c) Crystal packing arrangement showing interactions among 18-crown-6 ether, Cs^+, and L•SO_4^{2-} complex.

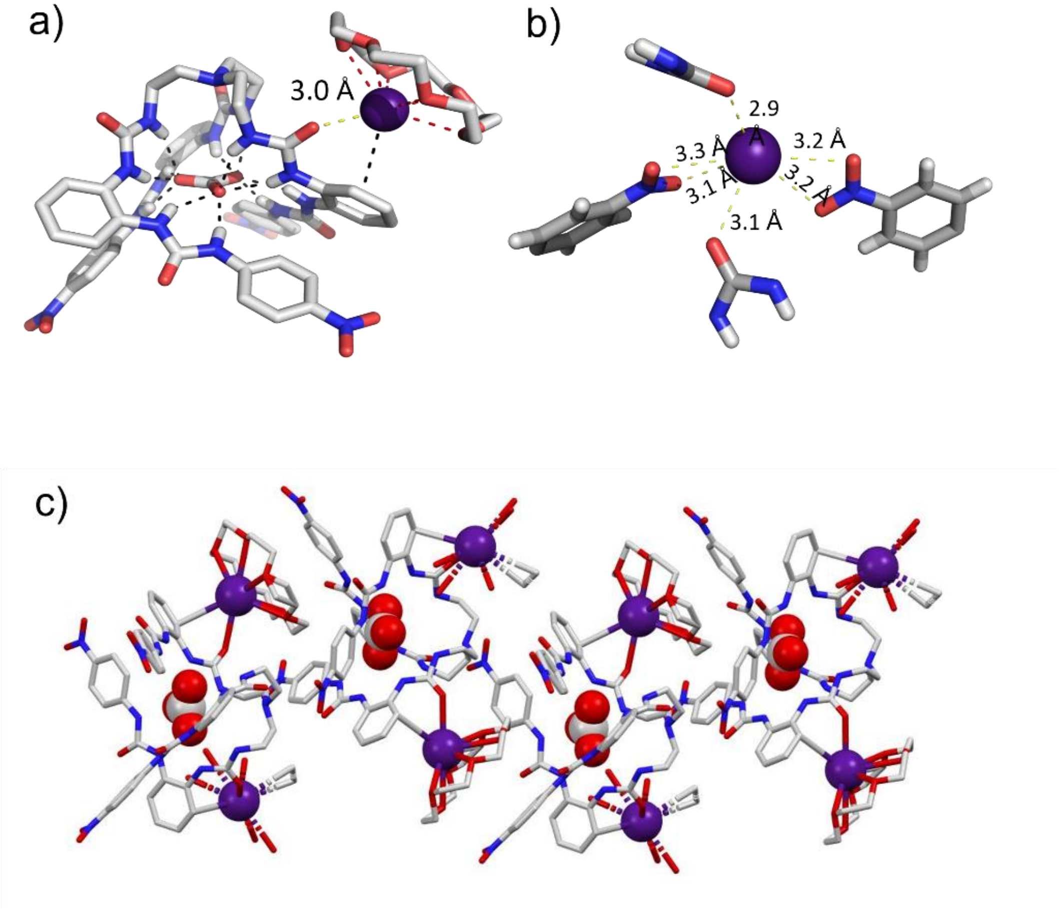

Figure S2. a) Single-crystal structure of the complex L•18-crown-6•Cs_2CO_3, the overall stoichiometry of Cs^+, CO_3^{2-}, 18-crown-6 and anion receptor is 2:1:1:1. b) Cs enhances the structural stability by interacting with the adjacent ureido carbonyl oxygen atom and the terminal nitro group oxygen atom. c) Crystal packing arrangement showing interactions among 18-crown-6 ether, Cs^+, and L•CO_3^{2-} complex.

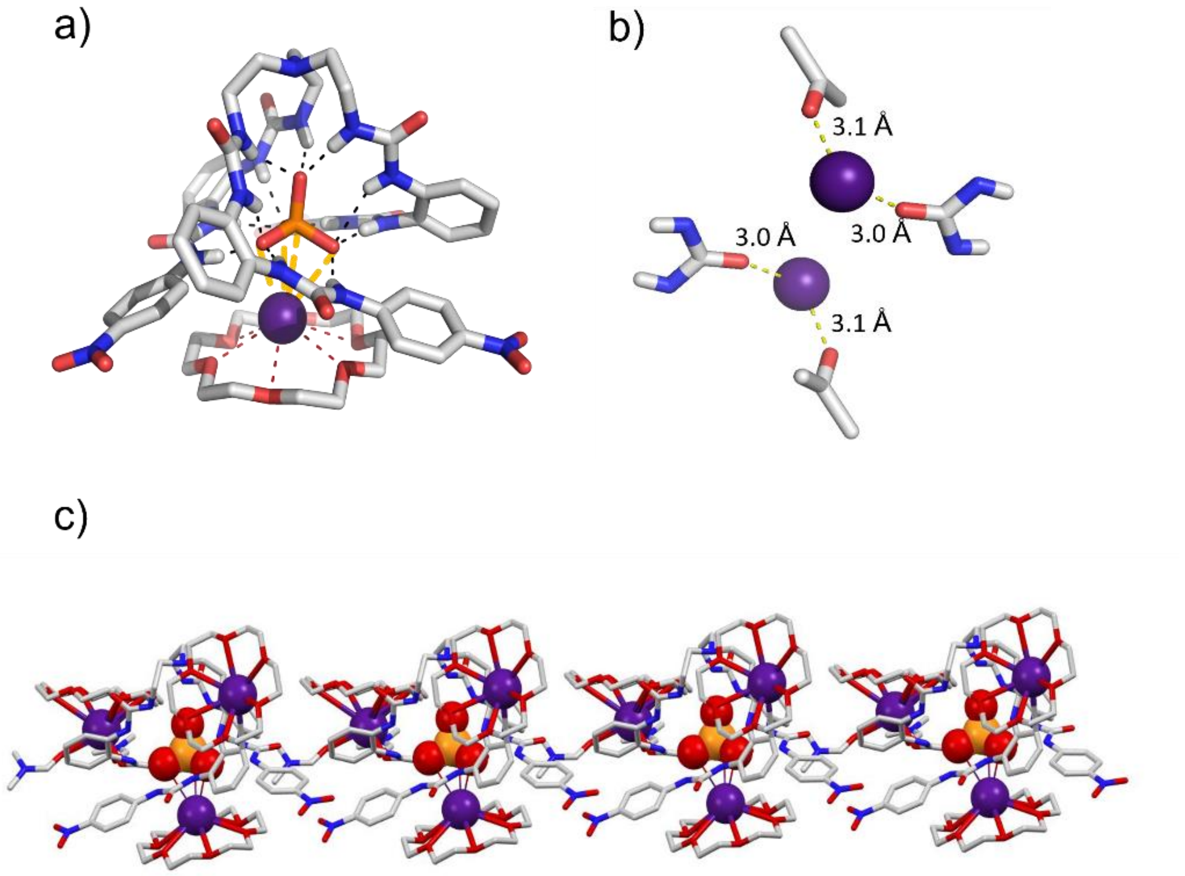

Figure S3. a) Single-crystal structure of the complex L•18-crown-6•Cs_3PO_4, the overall stoichiometry of Cs^+, PO_4^{3-}, 18-crown-6 and anion receptor is 3:1:3:1. One oxygen atom of the phosphate group points towards the nitrogen atom of the TREN, with three oxygen atoms pointing downwards to form a tetrahedron. b) Cs enhances structural stability by interacting with the ureido carbonyl oxygen atoms and acetone oxygen atoms. c) Crystal packing arrangement showing interactions among 18-crown-6 ether, Cs^+, and L•PO_4^{3-} complex.

S3. ^{1}H NMR titration studies

For a slow titration, such as titrating a ligand by adding carbonate anion as tetrabutylammonium salt (TBA_2CO_3), the equilibrium reaction equation can be represented as follows:

$$L + CO_3^{2-} \rightleftharpoons L \cdot CO_3^{2-} \qquad (1)$$

For the calculation, we define these:

$$x = [L \cdot (CO_3^{2-})] + [CO_3^{2-}]$$

$$y = [L \cdot (CO_3^{2-})]$$

$$a = [L]$$

the competitive equilibrium constant K can be represented as

$$K = \frac{[L \cdot (CO_3^{2-})]}{[L \cdot (CO_3^{2-})] \cdot [CO_3^{2-}]} = \frac{y}{(a-y) \cdot (x-y)} \qquad (2)$$

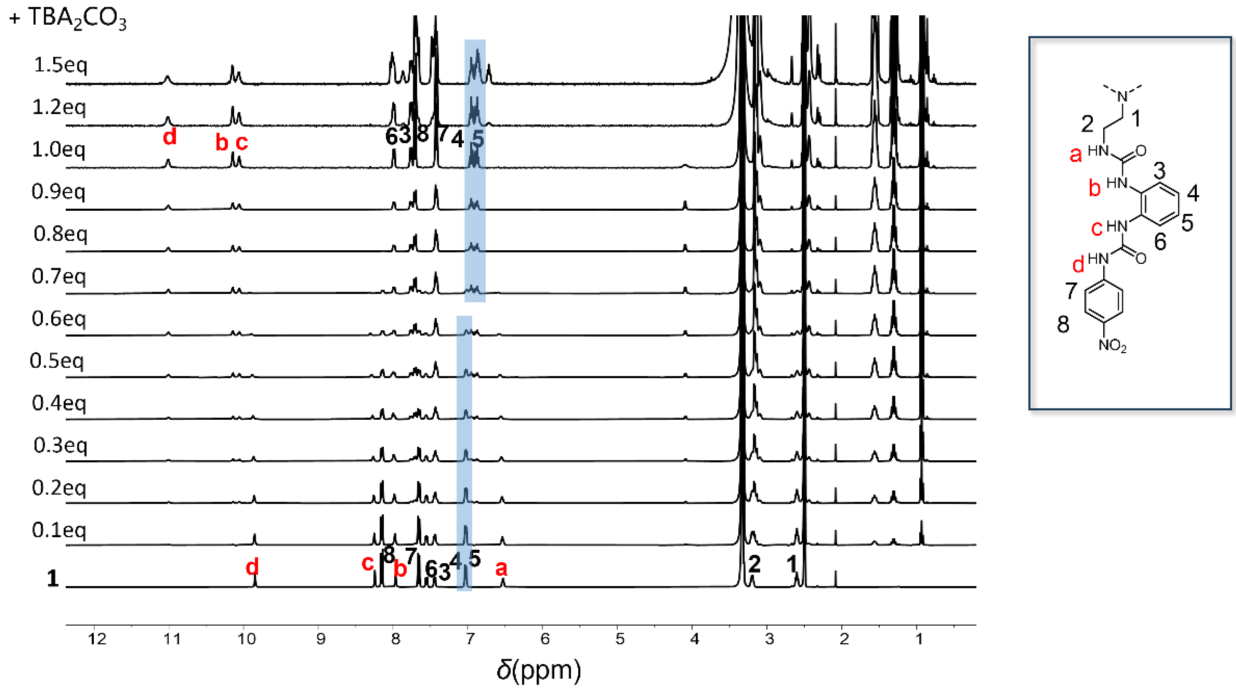

Figure S4. Stacked ^{1}H NMR spectra (400 MHz, 298 K, DMSO-d_6) of receptor L (2 mM) by adding carbonate anion as tetrabutylammonium salt (TBA_2CO_3, 50 mM).

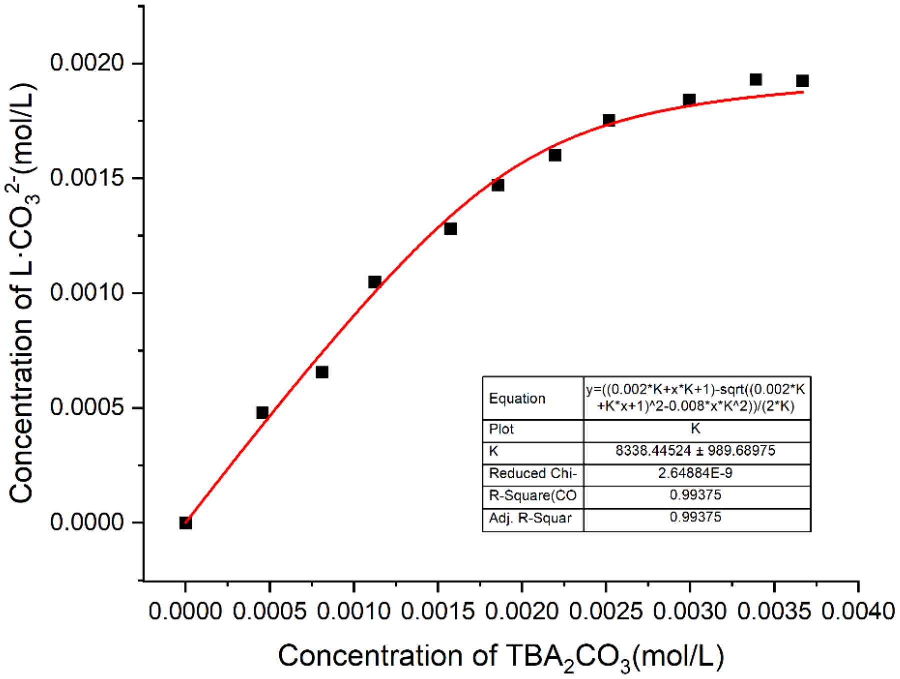

Figure S5. Fitted binding curve for the anion receptor by adding carbonate salts as determined by using the above-mentioned equation (2).

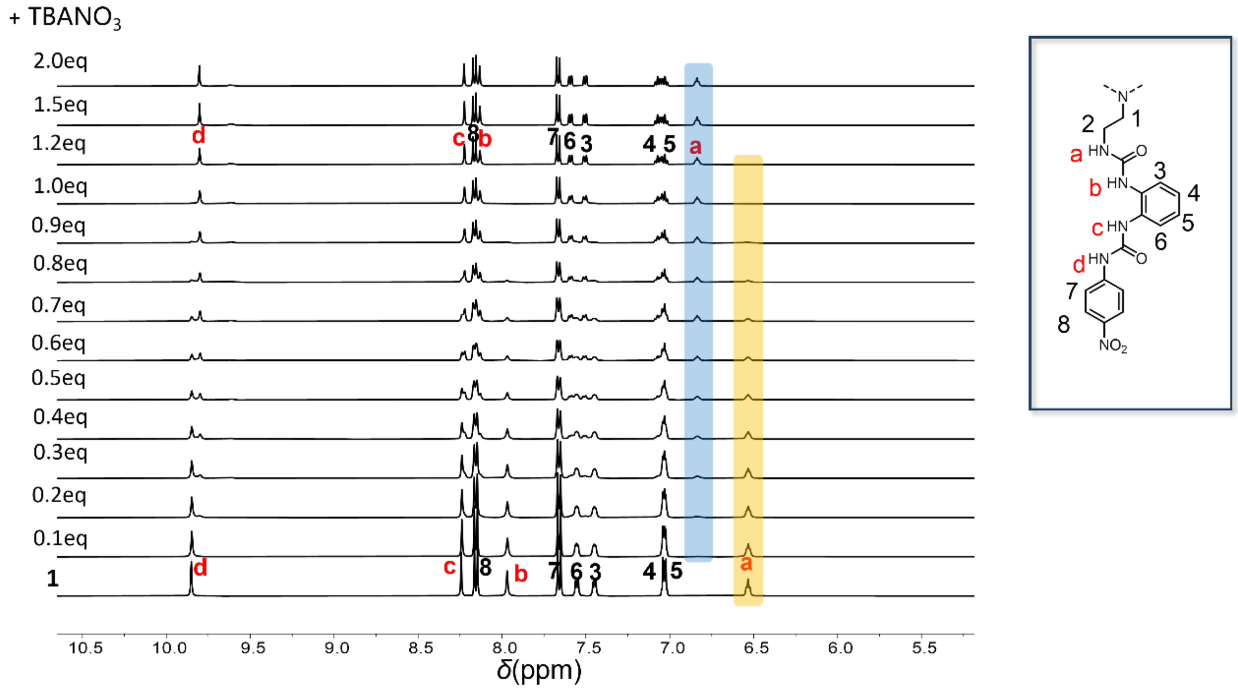

Figure S6. Stacked ^{1}H NMR spectra (500 MHz, 298 K, DMSO-d_6) of receptor L (2 mM) by adding nitrate anion as tetrabutylammonium salt ($TBANO_3$, 50 mM).

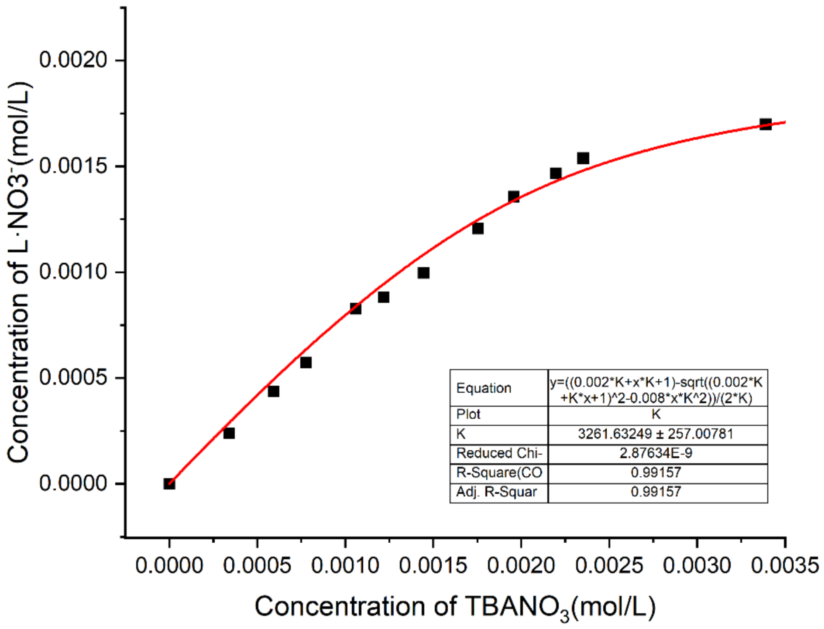

Figure S7. Fitted binding curve for the anion receptor by adding nitrate salts as determined by using the above-mentioned equation (2).

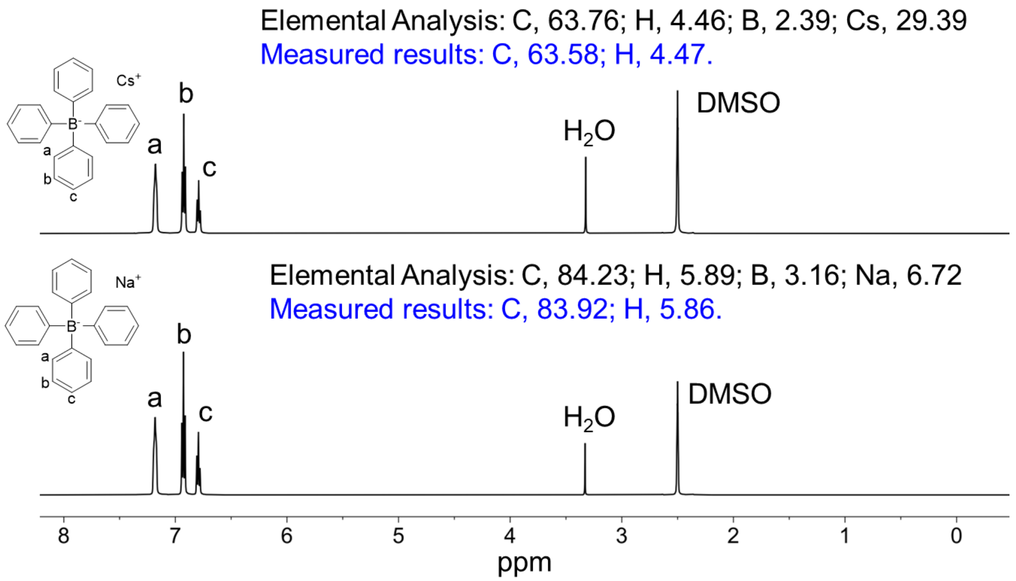

Figure S8. Stacked ^{1}H NMR spectra of cesium and sodium tetraphenylborate (1 mM, 400 MHz, DMSO-d_6, 298 K). As for the negligible ^{1}H NMR signal changes, elemental analysis experiments were conducted suggesting clear changes in C and H populations (color in blue).

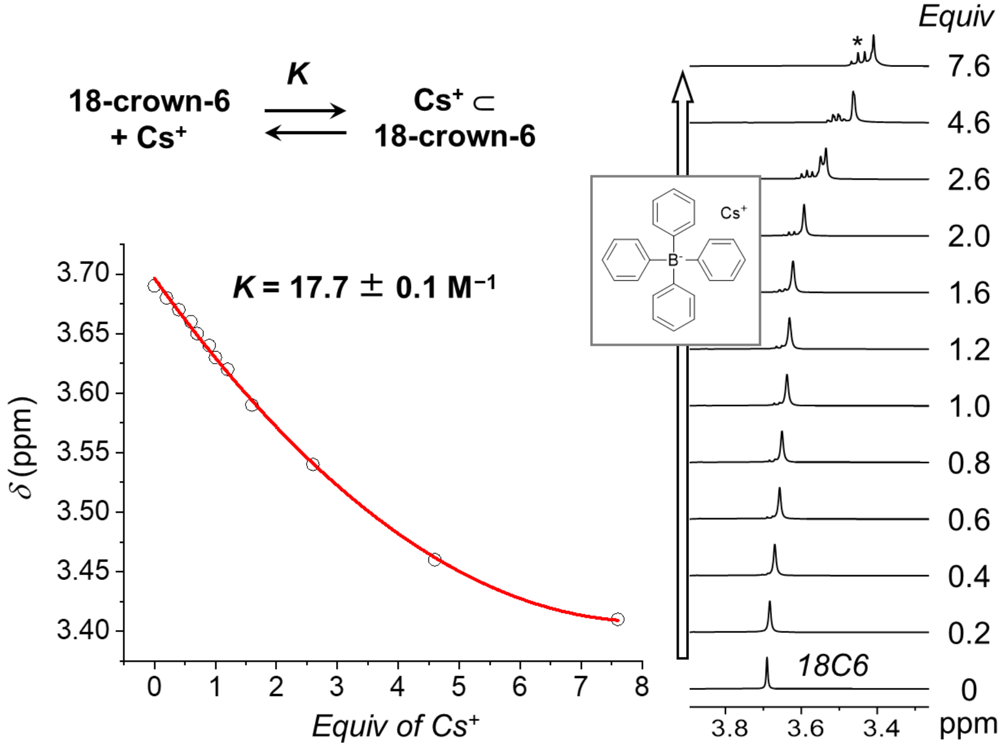

Figure S9. Stacked ^{1}H NMR spectra of 18-crown-6 by adding cesium tetraphenylborate salts (1 mM, 400 MHz, $CDCl_3$, 298 K) and its fitted binding curve as determined from online Bindfit website, http://app.supramolecular.org/bindfit/view/246be877-40d2-43d1-9067-371315f4428e. The calculated binding constants of free 18-crown-6 with Cs^+ cation is 17.7 ± 0.1 M^{-1}. * indicates residual ethyl ether. It is noted that the determined cesium binding affinity is clearly weaker than previously reported results (> 10^4 M^{-1} in CH_3CN, Elizabeth, M. et al., *J. Phys. Chem.* **1977**, 81, 1677–1681; Takeda, Y. *Bull. Chem. Soc. Jpn.* **1981**, *54*, 3133-3136.). This is likely due to that reverse titration (adding 18-crown-6 into the solution of $CsBPh_4$) is conducted before, where the 2:1 complex may form in acetonitrile and give a much stronger binding affinity.

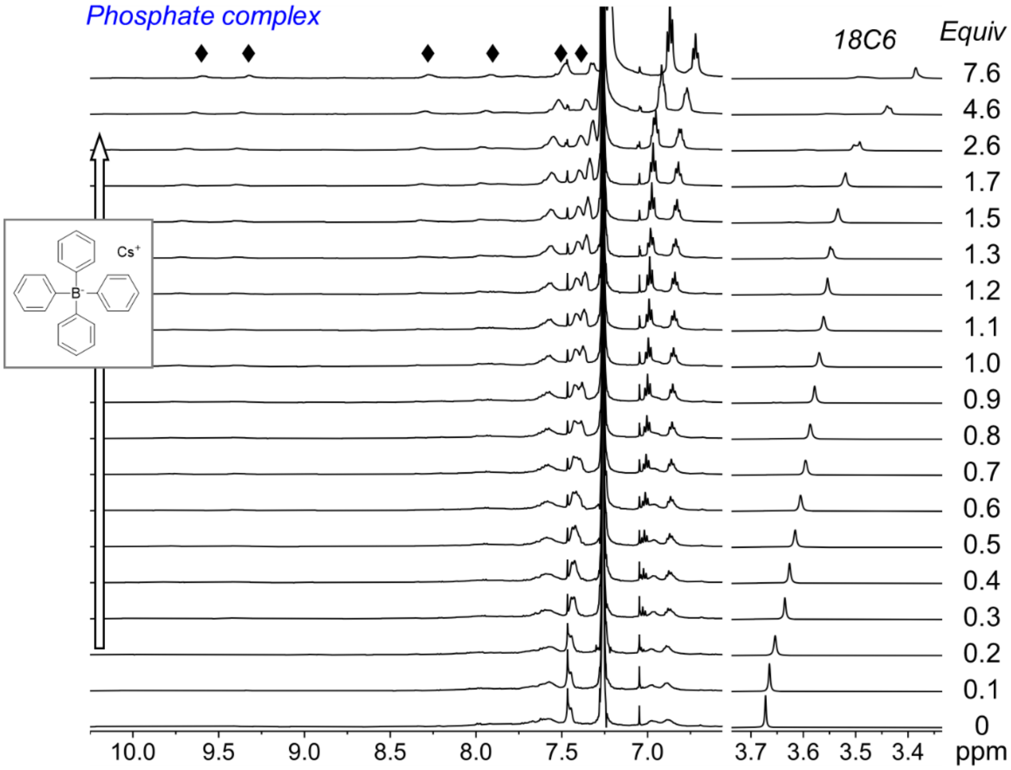

Figure S10. Stacked ^{1}H NMR spectra of 18-crown-6 by adding cesium tetraphenylborate salts with the presence of one equivalent of phosphate-hexaurea complex (1 mM, 400 MHz, $CDCl_3$, 298 K). Tetrabutylammonium phosphate salts were used. ♦ indicates the phosphate-hexaurea complex.

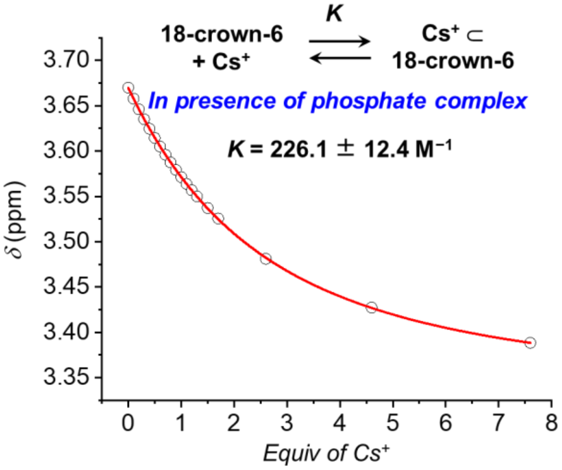

Figure S11. Fitted binding curve for the Cs+ binding of 18-crown-6 with the presence of phosphate-hexaurea complex as determined from online Bindfit website, http://app.supramolecular.org/bindfit/view/d60a6112-7c2a-4a75-9685-919c6e40b8b1. The calculated binding constant is 226.1 ± 12.4 M^{-1}.

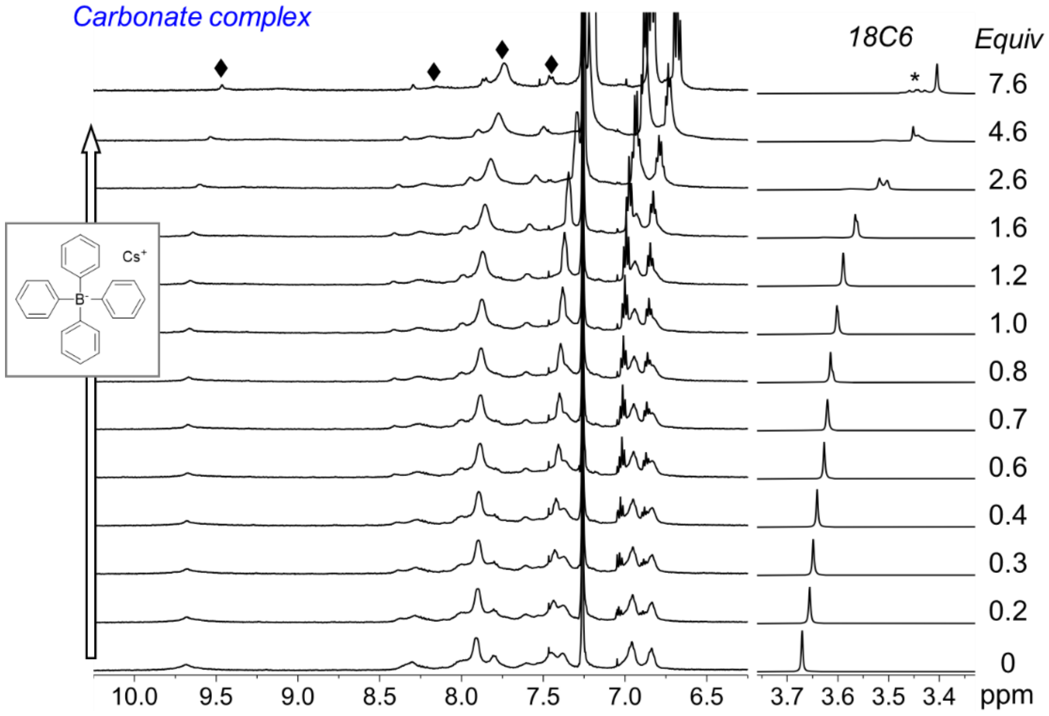

Figure S12. Stacked ^{1}H NMR spectra of 18-crown-6 by adding cesium tetraphenylborate salts with the presence of one equivalent of carbonate-hexaurea complex (1 mM, 400 MHz, $CDCl_3$, 298 K). Tetrabutylammonium carbonate salts were used. ♦ indicates the carbonate-hexaurea complex, * indicates residual ethyl ether.

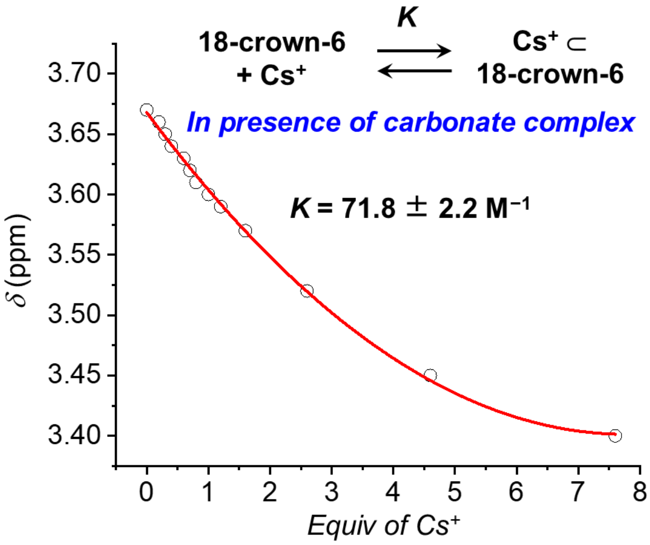

Figure S13. Fitted binding curve for the Cs+ binding of 18-crown-6 with the presence of carbonate-hexaurea complex as determined from online Bindfit website, http://app.supramolecular.org/bindfit/view/2b59538e-ce44-45a9-8abf-571f839e9e8e. The calculated binding constant is 71.8 ± 2.2 M^{-1}.

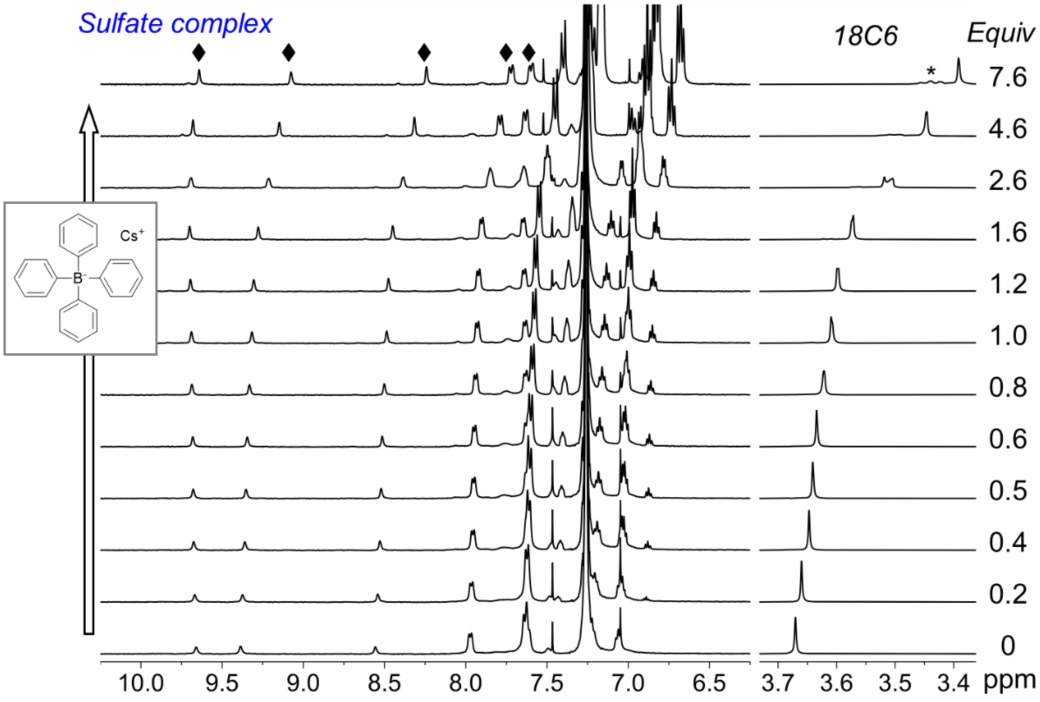

Figure S14. Stacked ^{1}H NMR spectra of 18-crown-6 by adding cesium tetraphenylborate salts with the presence of one equivalent of sulfate-hexaurea complex (1 mM, 400 MHz, $CDCl_3$, 298 K). Tetrabutylammonium sulfate salts were used. ♦ indicates the sulfate-hexaurea complex, * indicates residual ethyl ether.

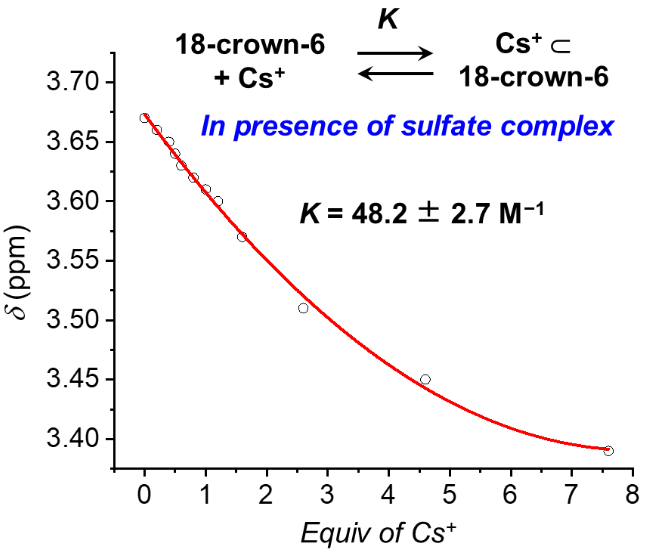

Figure S15. Fitted binding curve for the Cs+ binding of 18-crown-6 with the presence of sulfate-hexaurea complex as determined from online Bindfit website, http://app.supramolecular.org/bindfit/view/84b55e2c-5f66-433a-b9d6-74962d8d42c4. The calculated binding constant is 48.2 ± 2.7 M^{-1}.

S4. Solid-liquid extraction experiments

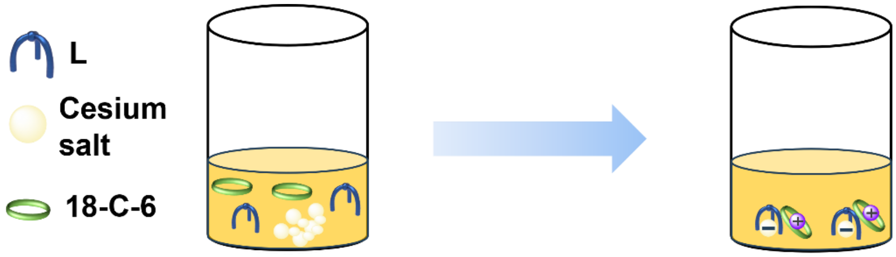

Figure S16. Diagram for solid-liquid extraction.

General solid–liquid extraction procedure: to a the acceptor solution (4.5 mM, 10 mL, $CHCl_3$), adding alkali metal cation solids (such as CsCl, $CsNO_3$, Cs_2CO_3, Cs_2SO_4, Cs_3PO_4). The added amount of cesium salts contains equivalent amount of anion by comparing to the hexaurea receptor. The solution is a yellow suspension. Mixing the prepared solution mixture and starting at 30 °C for 0.5 hours at a stirring speed of 1500 r/min. The solution and the insoluble solid are separated by centrifugation. The organic layer is carefully collected and dissolved in DMSO-d_6 for ^{1}H NMR analysis. The bottom sediment is washed with regular $CHCl_3$ (2 mL × 3) and redissolved in water (5 mL) by using a volumetric flask. An aqueous solution (0.5 mL) is taken and subjected to a 0.2 μM syringe filter, then the obtained aqueous solution is charged for ion chromatography to determine cation centration.

Table S5. Summary of IC results containing hexaurea receptor and 18-crown-6 for the solid–liquid extractions of cesium ions.

Cesium salts	Initial Cs^+ concentration(mM)	After extraction(mM)	Extraction efficiency
CsCl	4.33	0.79	81.8%
$CsNO_3$	5.16	3.29	36.2%
Cs_2CO_3	8.74	3.39	61.2%
Cs_2SO_4	8.03	4.76	40.8%
Cs_3PO_4	13.46	ND	100%

Table S6. The IC results of different cations extracted by the solid-liquid extraction method with the presence of hexaurea receptor and 18-crown-6.

Chloride salts	Initial Cl^- concentration(mM)	After extraction(mM)	Extraction efficiency
NaCl	5.75	3.19	44.5%
KCl	5.80	1.79	69.1%
CsCl	5.76	0.51	91.2%
LiCl	5.78	ND	100%

Table S7. Summary of IC results solid–liquid extraction of cesium ions by only adding anion receptor.

Cesium salts	Initial Cs^+ concentration(mM)	After extraction(mM)	Extraction efficiency
CsCl	4.33	3.88	10.4%
$CsNO_3$	5.16	4.48	13.2%
Cs_2CO_3	8.74	7.87	10.0%
Cs_2SO_4	8.03	7.72	3.9%
Cs_3PO_4	13.46	12.78	5.0%

Table S8. Summary of IC results solid–liquid extractions of cesium ions by only adding 18-crwon-6.

Cesium salts	Initial Cs^+ concentration(mM)	After extraction(mM)	Extraction efficiency
CsCl	4.33	1.40	67.7%
$CsNO_3$	5.16	3.56	30. 9%
Cs_2CO_3	8.74	5.37	38.6%
Cs_2SO_4	8.03	6.75	16.0%
Cs_3PO_4	13.46	5.58	58.5%

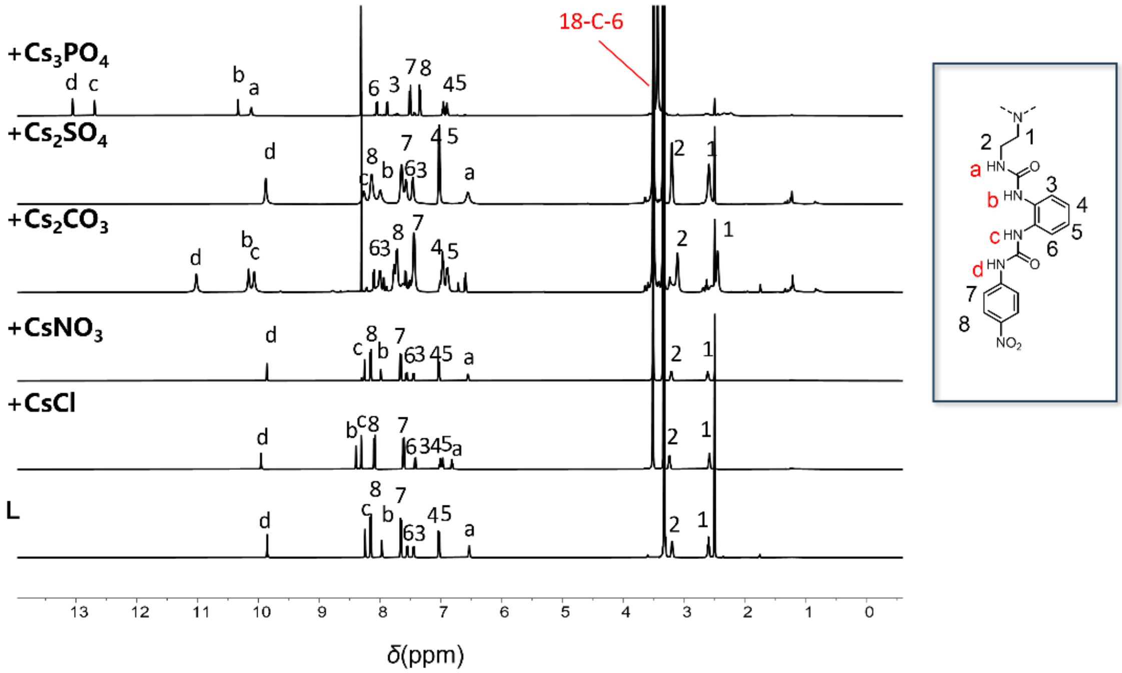

Figure S17. Stacked ^{1}H NMR spectra of free hexaurea receptor recorded in the absence and presence of CsCl, $CsNO_3$, Cs_2CO_3, Cs_2SO_4, Cs_3PO_4 in DMSO-d_6 (400 MHz, 298 K). All spectra were recorded after allowing the solid phase and the organic phase to equilibrate for 0.5 h.

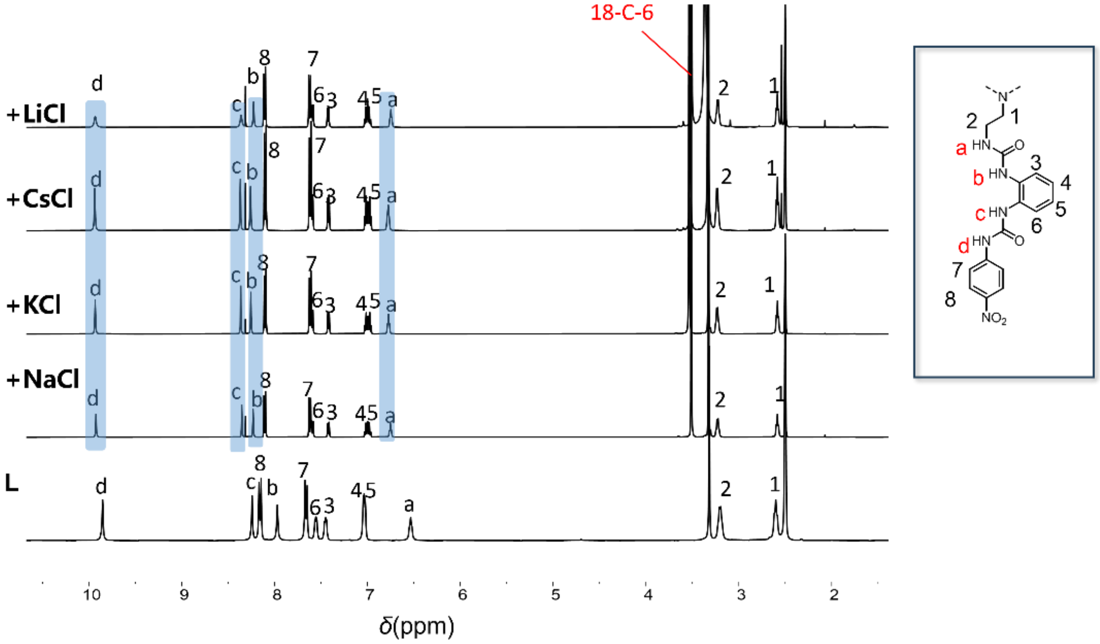

Figure S18. Stacked ^{1}H NMR spectra of free hexaurea receptor recorded in the absence and presence of LiCl, CsCl, KCl, NaCl in DMSO-d_6 (400 MHz, 298 K).

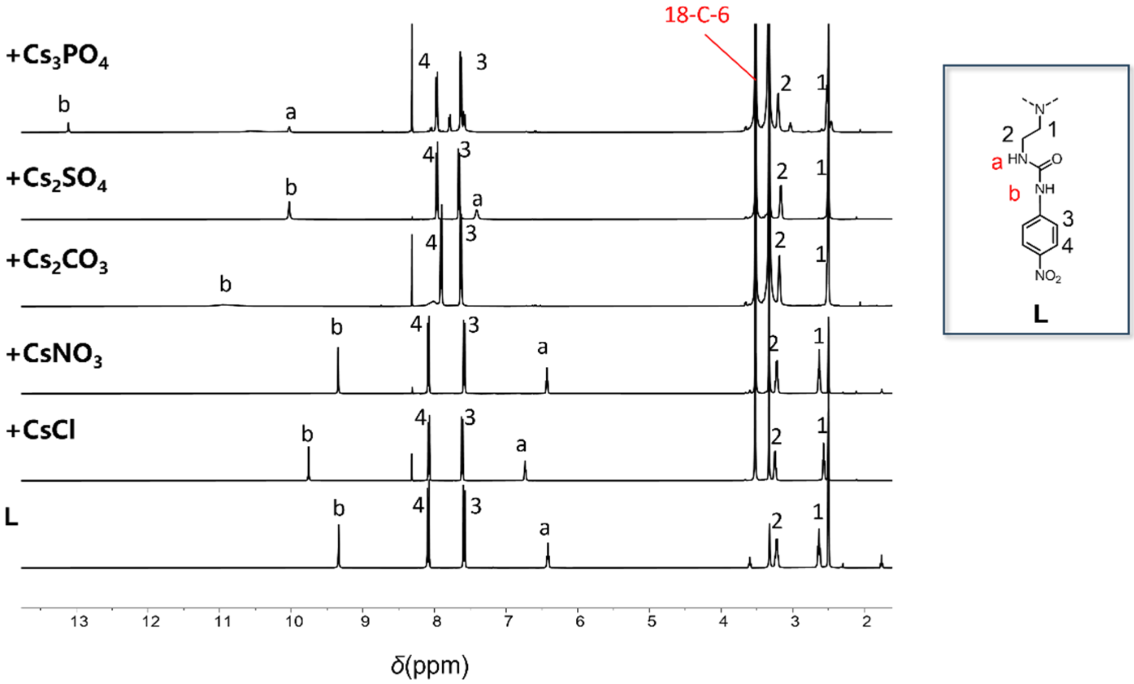

Figure S19. Stacked ^{1}H NMR spectra of free trisurea receptor recorded in the absence and presence of CsCl, $CsNO_3$, Cs_2CO_3, Cs_2SO_4, Cs_3PO_4 in DMSO-d_6 (400 MHz, 298 K).

S5. Mass spectrometry

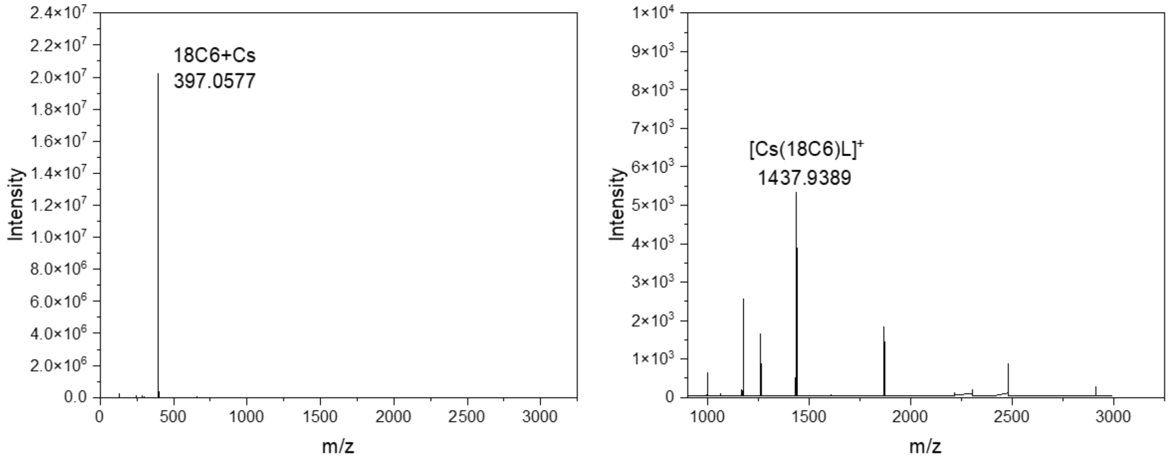

Figure S20. Full and enlarged ESI-MS spectra of [L • (18C6) • CsCl].

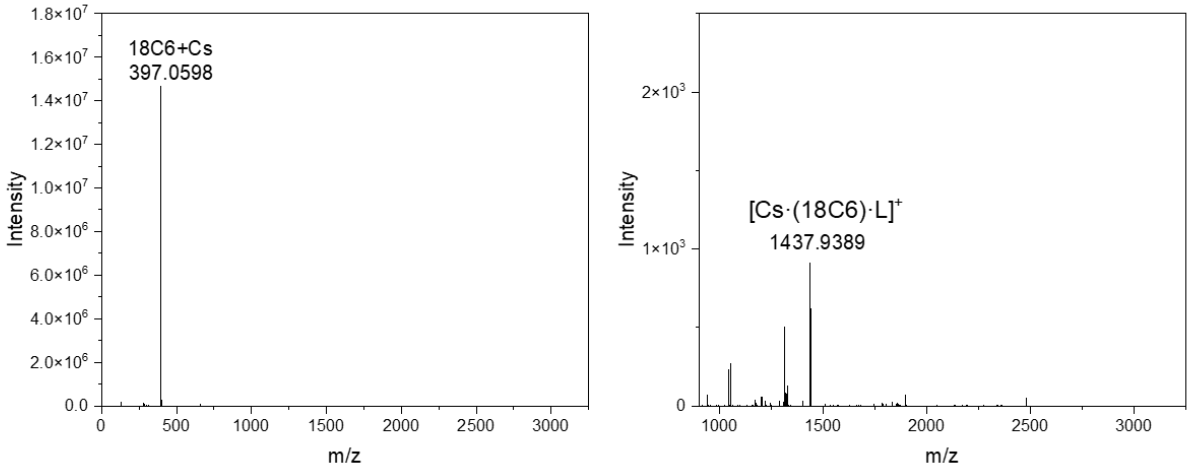

Figure S21. Full and enlarged ESI-MS spectra of [L • (18C6) • $CsNO_3$].

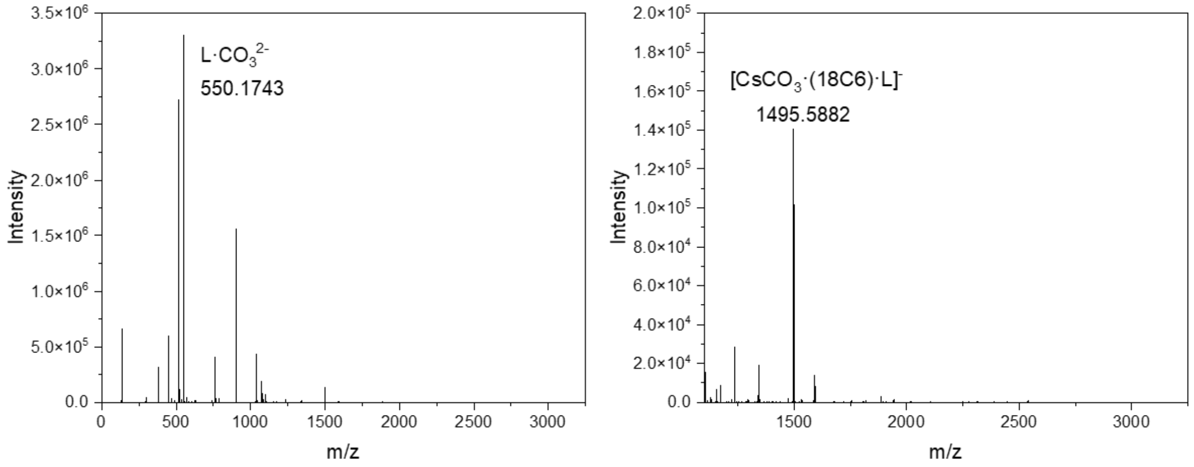

Figure S22. Full and enlarged ESI-MS spectra of [L • (18C6) • Cs_2CO_3].

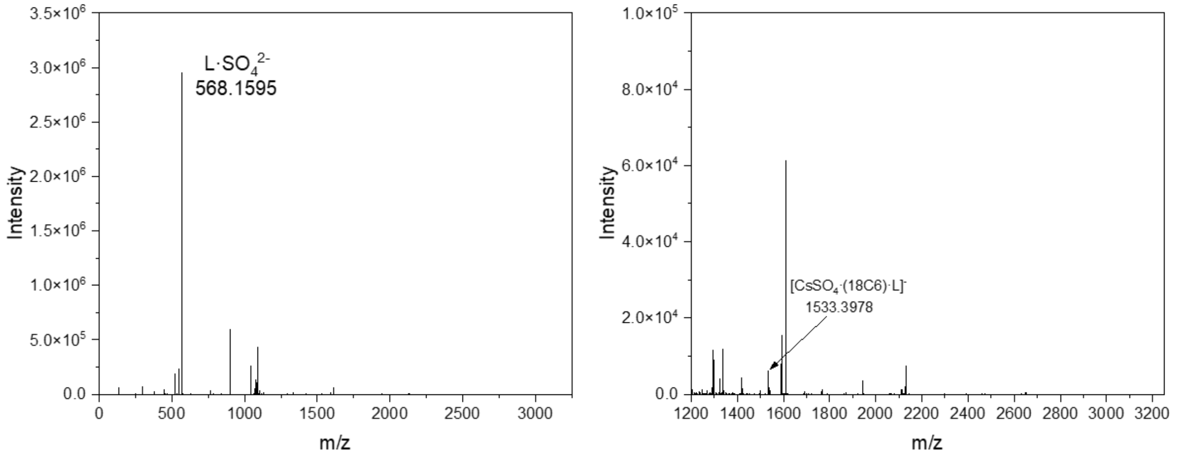

Figure S23. Full and enlarged ESI-MS spectra of [L • (18C6) • Cs_2SO_4].

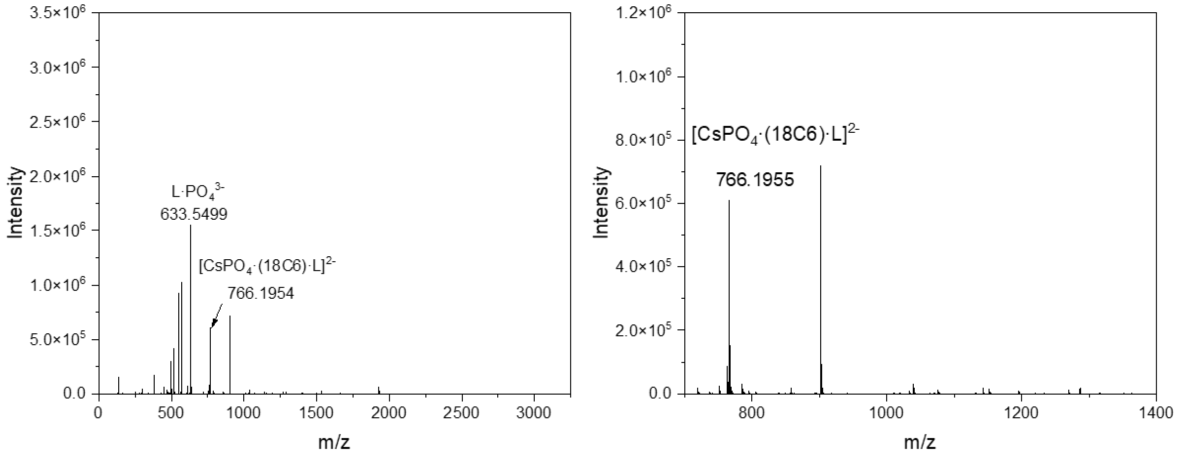

Figure S24. Full and enlarged ESI-MS spectra of [L • (18C6) • Cs_3PO_4].

S6. IGM analysis

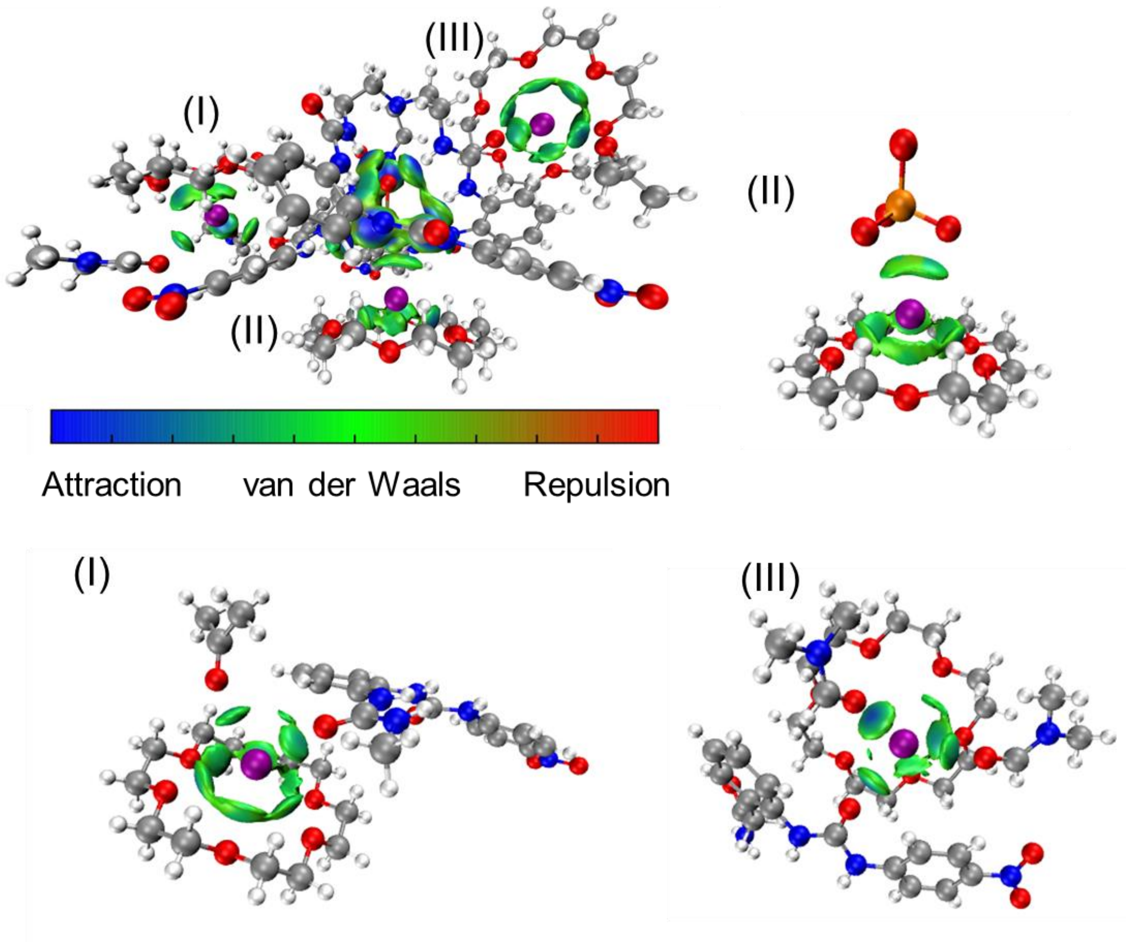

Figure S25. IGM plot of the Cs_3PO_4 complex illustrating that medium attraction between 18-crown-6 complexed Cs^+ and the phosphate complex. Color coding in the range of $-0.5 < \rho\ \mathrm{sign}(\lambda 2) < 0.5$ a.u. Atom colors: green or grey = C, white = H, blue = N, red = O, orange = P, and purple = Cs.

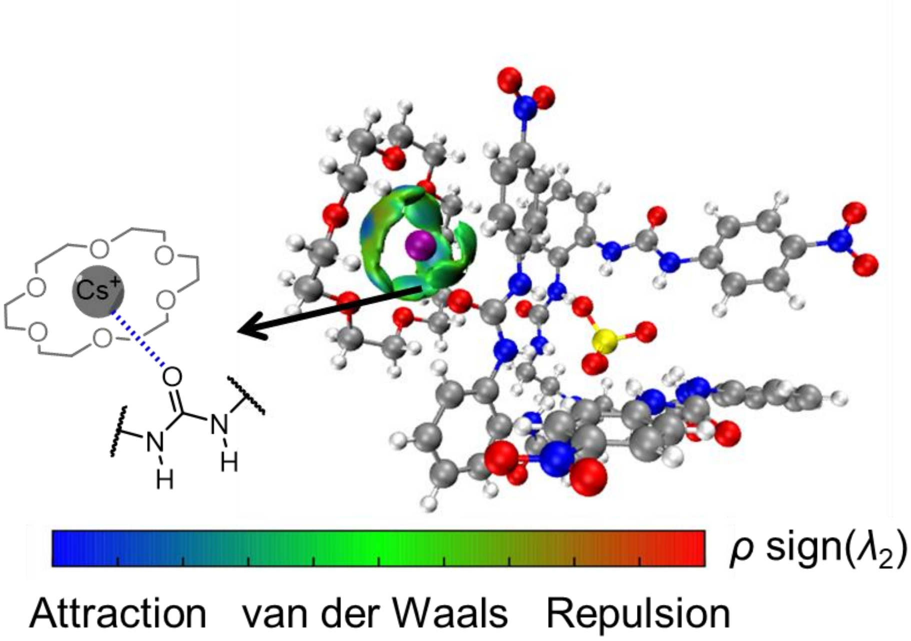

Figure S26. IGM plot of the Cs_2SO_4 complex illustrating that medium attraction between 18-crown-6 complexed Cs^+ and the O=C unit from the sulfate complex. Color coding in the range of $-0.5 < \rho$ sign(λ2) < 0.5 a.u. Atom colors: green or grey = C, white = H, blue = N, red = O, yellow = O, and purple = Cs.
